# A Multielectrode Array-Based Recording System for Analyzing Ultrasound-Driven Neural Responses in Brain Slices *in vitro*

**DOI:** 10.3389/fnins.2022.824142

**Published:** 2022-02-22

**Authors:** Ryo Furukawa, Hiroki Kaneta, Takashi Tateno

**Affiliations:** ^1^Graduate School of Information Science and Technology, Hokkaido University, Sapporo, Japan; ^2^Faculty of Information Science and Technology, Hokkaido University, Sapporo, Japan

**Keywords:** numerical simulation, multielectrode array, recording system, response pattern, ultrasound stimulation, waveguide design

## Abstract

Ultrasound stimulation is expected to be useful for transcranial local and deep stimulation of the brain, which is difficult to achieve using conventional electromagnetic stimulation methods. Previous ultrasound stimulation experiments have used various types of acute *in vitro* preparations, including hippocampus slices from rodents and Caenorhabditis elegans tissue. For *in vivo* preparations, researchers have used the cortices of rodents as targets for transcranial ultrasound stimulation. However, no previous studies have used *in vitro* ultrasound stimulation in rodent cortical slices to examine the mechanisms of ultrasound-driven central neural circuits. Here we demonstrate the optimal experimental conditions for an *in vitro* ultrasound stimulation system for measuring activity in brain slices using a multielectrode array substrate. We found that the peak amplitudes of the ultrasound-evoked cortical responses in the brain slices depend on the intensities and durations of the ultrasound stimulation parameters. Thus, our findings provide a new *in vitro* experimental setup that enables activation of a brain slice *via* ultrasound stimulation. Accordingly, our results indicate that choosing the appropriate ultrasound waveguide structure and stimulation parameters is important for producing the desired intensity distribution in a localized area within a brain slice. We expect that this experimental setup will facilitate future exploration of the mechanisms of ultrasound-driven neural activity.

## Introduction

Brain stimulation methods are used as clinical treatments for brain diseases and disorders including Parkinson’s disease and depression (Fregni and Pascual-Leone, 2007; Cardenas-Rojas et al., 2020). Among existing brain stimulation methods, transcranial ultrasound stimulation is minimally invasive, and this technique is expected to permit transcranial deep stimulation of the brain with a high spatial resolution, unlike conventional electromagnetic brain stimulation methods (for review, see Bystritsky et al., 2011; Blackmore et al., 2019; Pasquinelli et al., 2019). Uncovering the optimal stimulation conditions and relevant mechanisms will be necessary to enable transcranial ultrasound brain stimulation with maximal efficacy. Transcranial ultrasound brain stimulation has several drawbacks, including the induction of unintended sound perception in animal applications *in vivo*. Avoiding unexpected sound perception is particularly essential for transcranial ultrasound stimulation applications that aim to directly drive activity in the auditory central nervous system (Guo et al., 2018; Sato et al., 2018). The ability to reduce indirect activity propagation to a targeted area makes *in vitro* preparations and recordings particularly useful for examining the mechanisms of ultrasound-evoked neural activity in brain tissue.

Previous ultrasound stimulation experiments have used various types of acute *in vitro* preparations, including hippocampus slices from rodents (Tyler et al., 2008; Choi et al., 2013; Kim et al., 2017), isolated salamander retina (Menz et al., 2013, 2019), and Caenorhabditis elegans tissue (Kubanek et al., 2018). For *in vivo* preparations, researchers have used the cortices of rodents including mice (Bian et al., 2021), rats (Sato et al., 2018; Liu et al., 2021), and guinea pigs (Sato et al., 2018) as targets for transcranial ultrasound stimulation. However, to the best of our knowledge, no studies have used ultrasound *in vitro* stimulation in rodent cortical slices to examine the mechanisms of ultrasound-driven central neural circuits. We selected *in vitro* cortical slices as the experimental preparation in the present study because this system is effective for the delivery of ultrasound information, as well as for assessing the neural responses to ultrasound stimulation. Furthermore, cortical slice preparations enable visualization of the six cortical layers and anatomical organization while maintaining much of the cortical laminar structure and intrinsic neural circuitry (Namikawa et al., 2017; Yamamura et al., 2017; Muramatsu et al., 2019).

In this study, we measured activity evoked by ultrasound stimulation to *in vitro* brain slices that included the auditory cortex, with no neural inputs from the peripheral auditory system or other indirect propagation of activity. The choice of the biological preparation aims at a future medical application to hearing disorders. Furthermore, we searched for experimental conditions in which effective stimulation of brain slices was possible utilizing localized ultrasound wave irradiation. Once we identified the appropriate measurement equipment [e.g., an ultrasound transducer, multielectrode array (MEA) probe, perfusion system, and so on] and parameters for brain slice collection, the waveguide was the remaining adjustable component of the measuring system. Therefore, in this study, we mainly focused on the design of a waveguide, which we numerically and experimentally evaluated. To this end, we first numerically designed and examined a physical model of a waveguide combined with an ultrasound transducer to match the focus and spatial resolution of existing methods for ultrasound stimulation of a brain slice on an MEA substrate. Thus, we explored optimal experimental conditions for an *in vitro* ultrasound stimulation system for measuring activity in brain slices. Second, on the basis of the numerical results, we produced a waveguide with optimized stimulation conditions and constructed a measurement system to drive brain slices. Third, using the measuring system with the produced waveguide, we administered ultrasound stimulation to brain slices under optimized stimulation conditions to verify the effectiveness of the numerical calculation results and waveguide. Fourth, we analyzed the characteristics of the ultrasound-induced activity patterns obtained *via* MEA-based recording, compared with current-driven response patterns of brain slices and numerically calculated pressure distributions of ultrasound stimulation. Finally, on the basis of the results obtained in this study, we discuss appropriate conditions for ultrasound stimulation *in vitro*, and propose an improved method for conducting *in vivo* ultrasound stimulation to directly activate neural tissue in future work.

## Materials and Methods

### Measurement System Overview

**FIGURE 1:**
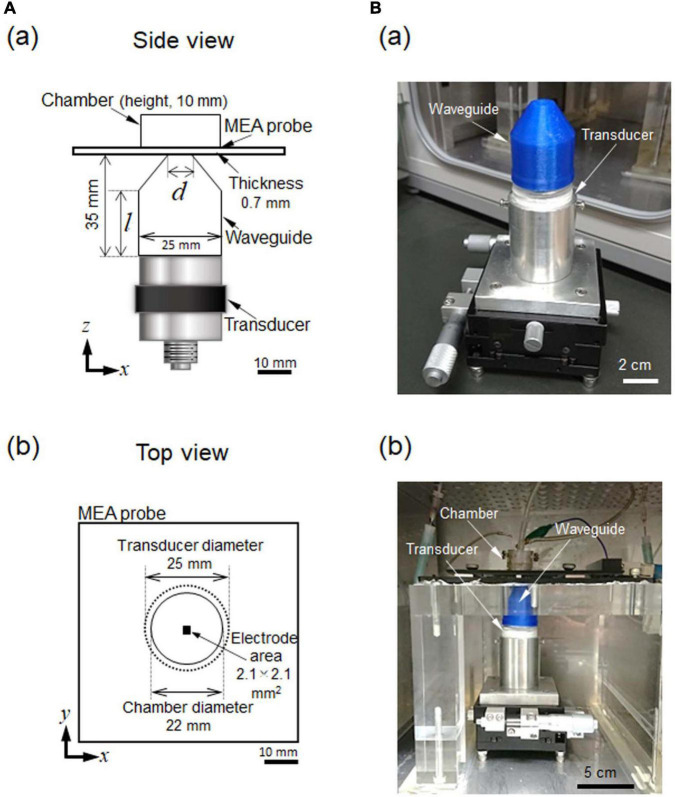
Ultrasound stimulation and MEA-based recording system combined with an incubator. **(A)** In (a), a schematic side view of the experimental setup. A waveguide (collimator) was combined with an ultrasound transducer. An MEA probe was located at the upper-most part of the recording system The optimal diameter (*d*) of the waveguide and the length (*l*) of the cylindrical part were determined *via* numerical simulation. The sizes of the other parts were predetermined according to the structural and physical constraints of the system. In (b), a schematic top view of the experimental setup. The recording area of the MEA probe was positioned in the center of the transducer and the waveguide. **(B)** In (a), an image showing the waveguide and transducer. These two parts are held in a fixture and supported on an *x*- and *y*-axis translation stage, which enable the center of the transducer to be finely moved in the horizontal *x*- and *y*- directions. In (b), an image illustrating the locations of the parts of the measurement system in (a) in the incubator. Recording cables and perfusion tubes can be seen on the upper stage.

We developed an *in vitro* experimental setup for measuring ultrasound stimulation-induced activity in mouse brain slices. We assumed that our measurement system could record extracellular potentials in brain tissue using an MEA substrate (Figure 1A). For extracellular recording, we conducted ultrasound irradiation using a transducer (V301-SU, Olympus, Waltham, MA, United States) with an MEA probe (MED-P530A, Alpha MED Scientific; glass substrate with a thickness of 0.7 mm and 64 microelectrodes in an area 2.1 × 2.1 mm^2^; Figure 1Ab). Each of the 64 recording sites (or channels; Chs) in the MEA covered an area of 50 × 50 μm^2^, and the interelectrode interval between the centers of adjacent sites was 300 μm. In the experiment, ultrasound waves were applied to a brain slice located on the glass substrate of an MEA probe. The waves were delivered from the transducer to the brain tissue through the bottom of the substrate (Figure 1Aa) *via* a custom-designed waveguide (collimator) (Figure 1Ba). Once we determined the size of the brain slices and the parameters of the measurement equipment, which included an ultrasound transducer, MEA probe, and perfusion system, the waveguide was the left as the only adjustable component in the measuring system. Therefore, in this study, we mainly focused on waveguide design by numerically and experimentally evaluating the waveguide properties.

When choosing the parameters for the ultrasound stimulation, we assumed that the focal length would need to be such that the stimulation would pass through glass MEA substrate (0.7 mm) and travel further to penetrate the brain slice (0.4 mm): i.e., 0.7–1.1 mm from the bottom edge of the MEA probe to the brain slice. Otherwise, the standing waves in the chamber containing the MEA probe would be dominant, and the power distribution in the chamber during ultrasound stimulation would be too complex for our analysis.

We calculated the average acoustic intensity (power) of the ultrasound pulse according to the acoustic pressure distribution of the ultrasound wave traveling through the waveguide (Figure 1A). We defined the spatial resolution as the length corresponding to more than half of the maximum ultrasound intensity. To determine the localization of the ultrasound intensity on the MEA substrate amongst the 64 electrodes, we set the spatial resolution (centered at the peak position) to be less than 2.1 mm. This was the size of the area covered by the multielectrode array and the central area of a typical brain slice, i.e., 3.5 × 4.0 mm^2^ (Figure 1Ab).

### Simulation of a Focal Area via Ultrasound Stimulation

To design the waveguide used to focus the ultrasound irradiation within a brain slice, we numerically calculated the spatial distribution of ultrasound intensities within the slice. We calculated the focal length from the transducer to the focal point using the waveguide located on top of the transducer. Because of the axial (cylindrical) symmetry around the center axis, we first created a two-dimensional (2D) simulation of the ultrasound propagation using the finite-difference time-domain (FDTD) method (Toda and Tateno, 2020). A three-dimensional (3D) simulation was also performed (as explained in the next subsection) to examine the 3D distribution of the ultrasound intensity.

Here, we explored the desired acoustic pressure field by searching for appropriate structural parameters regarding the length (*l*) and diameter (*d*) of the opening end of the waveguide (Figure 1A). Because of a size restriction in our recording system, the height of the waveguide was set at 35 mm (Figure 1Aa). Thus, the length *l* must be equal to or smaller than the height (i.e., *l* ≤ 35 mm).

In the 2D FDTD method, the governing equations of medium particle motion are described as:


(1a) $$\rho_{w}\frac{\partial v_{x}}{\partial t}=-\frac{\partial p}{\partial x}$$

(1b) $$\rho_{w}\frac{\partial v_{y}}{\partial t}=-\frac{\partial p}{\partial y}$$

where *ρ_*w*_* is water density (*ρ_*w*_* = 1.0 × 10^3^ kg/m^3^), *v*_*x*_ and *v*_*y*_ are the particle velocities of the medium in the *x* and *y* directions, respectively, *p* is pressure, and *t* is time. The continuous equation concerning pressure *p* is described as:


(2) $$\frac{\partial p}{\partial t}=-\kappa \left(\frac{\partial v_{x}}{\partial_{x}}+\frac{\partial v_{y}}{\partial y}\right)$$

where κ is the bulk modulus of the medium (κ = 2.19 × 10^9^ kg/m^2^s).

The edge of the computational area was conditioned as a free boundary, and the perfectly matched layer (PML) boundary (Chew and Liu, 1996) was used as a boundary condition (Eq. 3a). In addition, the boundary between the waveguide and the water was set as the sound-absorbing boundary. It was mounted using surface normal impedance *Z*_*n*_, described as:


(3a) $$v_{x}=-p/Z_{n},v_{y}=-p/Z_{n}$$

(3b) $$Z_{n}=\rho_{w}c_{w}\sqrt{1-a_{n}}/\sqrt{1+a_{n}}$$

where *c*_*w*_ is the sound velocity in water (*c*_*w*_ = 1.48 × 10^3^ m/s) and *a*_*n*_ is the sound absorption coefficient at normal incidence. The minus sign in Eq. 3a indicates the reversal of the particle velocity direction because of reflection. The spatial-discrete width Δ*h* was set to 1/20 of the wavelength (input wave number = 5) of the upper limit of the frequency to be analyzed; Δ*h* = 5.0 × 10^–5^ m. The time-discrete width was set to satisfy the Courant–Friedrichs–Lewy (CFL) condition, which was necessary for calculation stability. The coolant *C*, with the CFL condition as a dimensionless number, is described as:


(4) $$C=c_{w}△t/△h$$

where Δ*t* is the time-discrete width (Δ*t* = 5.0 × 10^–9^ s) because the coolant number is usually smaller than 1/$\sqrt{2}$ in 2D simulations.

**FIGURE 2:**
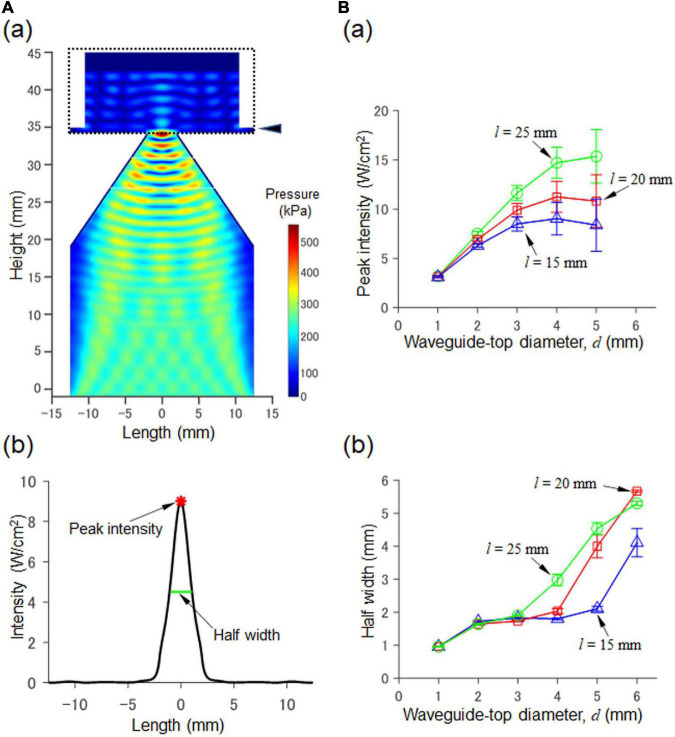
Numerical simulation of a 2D model consisting of a waveguide and an MEA probe. **(A)** In (a), the acoustic pressure distribution in the cross section passing through the central axis. The size parameters are *d* = 4.0 mm and *l* = 20 mm (Figure 1Aa). In (b), for the cross section at a height of 35.7 mm in (a) (indicated by the arrowhead on the upper right), the corresponding intensity (*I*_sppa_) calculated from the pressure is shown. In the plot, the peak intensity (red asterisk) and the halfwidth (green line) of the pressure are also indicated by arrows. **(B)** In (a), the results of our numerical simulation with respect to the relationship between the diameter *d* and peak intensity (*I*_sppa_) are summarized for fixed three values of *l* (i.e., *l* = 15, 20, and 25 mm; height 35.7 mm as in (a)). In (b), the relationship between the diameter *d* and the half width is illustrated for the three fixed values.

The simulation field area is illustrated in Figure 2. The area was surrounded by a rectangle of 50 × 100 mm^2^. The PML boundary was defined as the edges of the rectangle. The input ultrasound (at *y* = 0 in Figure 2A) was a sinusoidal wave (*f* = 500 kHz) that passed through the waveguide, which was filled with water. The transducer diameter was 25 mm in our experimental setup (Figures 1Ab,B). The waveguide sidewall was made of polypropylene (PP) with a sound absorption coefficient *a*_*n*_ of 0.876, calculated using the acoustic impedance between water and PP (Lochab et al., 2004). At the top of the waveguide, the bottom of the glass MEA substrate was attached to the surface of the waveguide, which effectively transmitted the power of the ultrasound waves (Figures 1Aa, 2Aa).

As an index of ultrasound intensity, we used the power integral intensity (spatial peak pulse average, *I*_sppa_), which was calculated from acoustic pressure *p*(*t*) and described as:


(5) $$I_{\text{sppa}}=\frac{1}{T}\int_{0}^{T}\frac{p^{2}\left(t\right)}{\rho_{w}c_{w}}dt,$$

where the time window *T* for the integral ranged from 0 to 125 ms (i.e., *T* = 125 ms). The maximum ultrasound intensity was analyzed using *I*_sppa_ (Eq. 5). The area (length in the 2D simulation) in which the intensity was more than the half of the maximum intensity was defined as the half-value area (length), and the longest width in the area (length) was defined as the half width (HW) (Figure 2Ab). When standing waves of pressure were observed, as was the case for some parameter sets, several local peaks appeared. In such cases, the half-value area (length) was obtained after calculating the envelope of the corresponding peak intensities. *In vitro*, when the amount of artificial cerebral spinal fluid (ACSF) solution in the MEA chamber changes, the height (*h*) of the fluid level changes, affecting the intensity distribution. Therefore, we varied the amount of ACSF solution such that the fluid level *h* increased from 1.0 to 10 mm in 1.0-mm steps. For numerical calculations, we used a parallel computer system (PRIMERGY CX400/CX2550, Fujitsu, Japan) at the Hokkaido University Computer Center. In addition, we used GNU Fortran 5.4.0 for the compiler and OpenMP for the parallel programming.

### 3D Simulation Using the Finite Element Method

**FIGURE 3:**
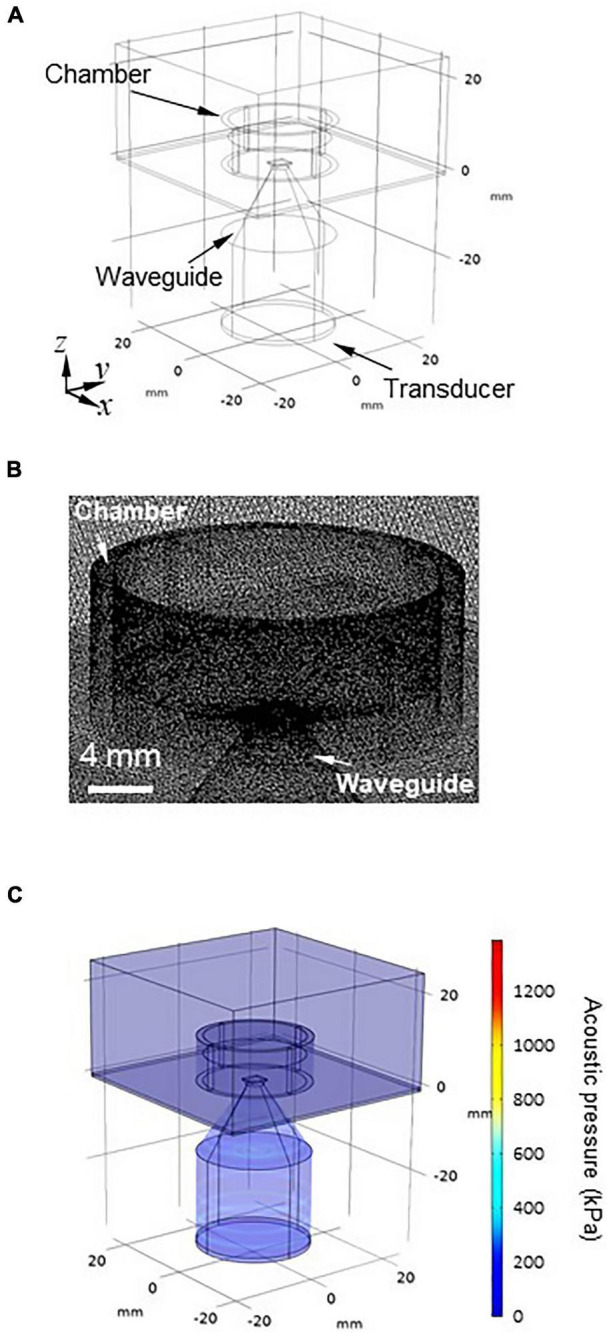
3D modeling for numerically obtaining the spatial pressure distribution. **(A)** Geometry of the 3D model. The 3D model has six parts: (i) transducer, (ii) waveguide, (iii) MEA probe with a glass camber, (iv) brain slice, (v) artificial cerebrospinal fluid (ACSF) solution, and (vi) air surrounding the five components. **(B)** Mesh image showing the space around the chamber of the MEA probe using model geometry. The geometry of the 3D model was discretized to the extra fine mesh (maximum = 1.23 mm, minimum = 0.123 mm). **(C)** Typical overview of the acoustic pressure distribution of the 3D model, which was numerically obtained for the size parameters *d* = 4.0 mm and *l* = 20 mm.

To confirm the results obtained from the 2D FDTD method under more practical conditions, we conducted a simulation with a 3D model using the finite element method (FEM). The simulation was performed using commercially available multiphysics software, COMSOL Multiphysics (ver. 5.5, COMSOL AB, Sweden). The 3D model consisted of a transducer, a waveguide, an MEA probe with a glass chamber, a brain slice, ACSF solution, and the air surrounding these components (Figure 3A). As with the 2D model, the amount of ACSF solution in the MEA chamber was varied in 10 levels (*h*) ranging from 1.0 to 10 mm in 1.0-mm steps.

The geometry of the model was discretized to the extra fine mesh (the maximum is 1.23 mm and the minimum is 0.123 mm) setting with a swept mesh for the infinite air domain (Figure 3B). The acoustic pressure at the surface of the transducer was set to 100 kPa (*I*_sppa_ = 0.34 W/cm^2^; for a definition of *I*_sppa_, see Eq. 5). The rectangular brain slice (4.0 × 3.5 × 0.4 mm^3^) was located on the surface of the MEA substrate, which had an acoustic impedance of 1,558 kPa⋅s/m. With respect to the pressure acoustics, the frequency domain-interface and laminar flow-interface in the software were used in the numerical calculations.

The boundary conditions of the sidewalls in the chamber and waveguide were the set using the “sound hard boundary” option in the program. The “sound soft boundary” was applied for the outer boundaries of the air domain and the boundary between the saline solution and air. We performed 3D modeling with the FEM using the same parallel computer system (PRIMERGY CX400/CX2550, Fujitsu, Japan) at the Hokkaido University Computer Center. In the model, the boundary conditions of the surrounding air domain were set to approximate infinity so that they minimally affected the numerical solution.

### Pressure Measurement Using a Hydrophone

To examine the irradiation properties of ultrasound waves traveling through our designed waveguide, we measured the acoustic pressure using a calibrated needle hydrophone (HY05N, Toray Engineering Co., Chuo, Japan) with a calibration certificate supplied by the manufacturer. The hydrophone was vertically lowered into the ACSF solution in the chamber of an MEA probe, and the measured data were recorded using an oscilloscope with a digital data acquisition system (SDS 1104X-E, SIGLENT Technologies, Shenzhen, China).

To characterize the waveguide properties, we used a continuous ultrasound waveform (CW) with a fixed window length (duration, 20 μs) to record the hydrophone output. We started recording once the signal had stabilized. Because of the central symmetricity, we measured pressure in one quadrant of the circular chamber filled with ACSF solution. To measure hydrophone output at different planar positions, the device was initially positioned at the approximate center of the MEA plane. Then, the hydrophone was repositioned in 0.5 mm increments to a maximum distance of 4.5 mm from the center on each axis. The distance between the ultrasound transducer and the hydrophone was set to approximately 1.0 mm. The distance was confirmed using sound velocity of the trigger delay time in distilled water or ACSF solution. The sound velocity was calculated as a function of temperature, and the temperature of water (or ACSF solution) was measured using a thermometer (RT-31S, ESPEC MIC CORP., Niwa, Japan). To place a brain slice on an MEA substrate, we used a nylon mesh and an anchor (see Section “Multielectrode-Array for Recording Activity in Brain Slices”). Because the nylon mesh was 1.1 mm mesh opening and 0.12 mm thick, there was little difference between our pressure measurements in the presence and absence of the mesh. Also, a U-shaped anchor was made from a 0.3-mm stainless wire and spacing by the edge distance of 8 mm. The anchor itself did not affect the pressure measurement on the area of multielectrodes (2.1 × 2.1 mm^2^).

For the CW, the original rectangular wave with a fundamental frequency of 500 kHz was generated by a function generator (WF1947, NF Co., Yokohama, Japan). In terms of the temporal window pattern, the CW stimuli had a duration of 100 or 200 ms. The acoustic pressure was varied from 110 to 410 kPa in 100 kPa steps: i.e., 110, 210, 310, and 410 kPa, and the corresponding power integral intensity (spatial peak pulse average, *I*_sppa_ in Eq. 5) was 0.41, 1.49, 3.24, and 5.68 W/cm^2^, respectively. The input voltage signals from the function generator to the transducer were all amplified with a 50-dB radio-frequency amplifier (ZSA5064, RAD Co., Fuji, Japan).

### Ultrasound Waveform Generation and Brain Slice Stimulation Patterns

As mentioned in the previous subsection, a 500-kHz ultrasound signal was delivered from a planar ultrasound transducer (V301-SU, Olympus) through the custom-designed waveguide attached to the transducer, which was positioned vertically below the MEA probe (at a 90-degree angle) (Figure 1A). The waveguide was optimized to activate a small region of a brain slice (HW ≤ 2.1 mm). This design helped minimize the generation of standing waves and created a favorable interface on the bottom of the MEA dish. Thus, it was possible to use this configuration to produce a controllable ultrasound intensity distribution. The custom-designed waveguide was then produced using a 3D printer (Dreamer, Flashforge, Zhejiang, China) with a polypropylene (PP) filament (1.75-mm diameter; Flashforge, Osaka, Japan). To align the properties of the transducer with those of the waveguide, the waveguide end was sealed with polyethylene, and the inside was filled with degassed water.

The acoustic frequency and intensity characteristics of an ultrasound waveform stimulus are essential elements of its core effects on brain activity. Waveform patterns can also influence the efficiency of neural activation. For our neural stimulation, we used a simple sinusoidal waveform: a CW signal with a fixed window length. For the CW, we generated an original rectangular wave with a fundamental frequency of 500 kHz using a function generator (WF1947). In terms of the temporal window pattern, the duration of the CW stimuli was 100 or 200 ms. The acoustic pressure varied from 110 to 410 kPa in 100 kPa steps: i.e., 110, 210, 310, and 410 kPa, such that the intensity (*I*_sppa_ in Eq. 5) corresponded to 0.41, 1.49, 3.24, and 5.68 W/cm^2^, respectively. The signals from the function generator were amplified using a 50-dB radiofrequency amplifier (ZSA5064).

### Multielectrode-Array for Recording Activity in Brain Slices

All animal experiments described below were approved by the Institutional Animal Care and Use Committee of Hokkaido University and carried out in accordance with the National Institutes of Health Guidelines for the Care and Use of Laboratory Animals. In the present study, we used five C57BL/6J mice (three male and two female mice, 4–8 weeks old, Japan SLC Inc., Hamamatsu, Japan). Each mouse was deeply anesthetized with isoflurane and decapitated. When sectioning brain slices that included the auditory cortex, the distance from the bregma along the rostral/caudal axis in each coronal section was defined in the following way. We referred to a digitized atlas (Franklin and Paxinos, 2008) for illustrations of coronal sections of the mouse brain. We chose the coronal section that best matched the illustration at 2.92 mm caudal to bregma (Figure 5 in Franklin and Paxinos, 2008). Coronal 400-μm-thick slices that included the auditory cortex were cut using a tissue slicer (Linear Slicer Pro7, D.S.K., Kyoto, Japan) in chilled ACSF. The ACSF contained (in mM) 119 NaCl, 2.5 KCl, 2.5 CaCl_2_, 1.3 MgSO_4_, 1.0 NaH_2_PO_4_, and 11.0 D-glucose (pH = 7.4). All slices were left in a submerged-type holding chamber at 28°C in a water bath for at least 2 h before recording.

To record ultrasound-driven local field potentials (LFPs) from mouse brain slices *in vitro*, we developed a custom *in vitro* ultrasound stimulation system that we combined with an MEA-based recording system (Figure 1A). All electrophysiological recordings in brain slices were performed with perfusion of ACSF solution with a stable flow rate of 1.26 ± 0.01 ml/min. During the recording, the flow rate was nearly constant, such that we expected the height of the ACSF solution in the MEA chamber to be unchanged. During the perfusion, mixed gas was supplied from the top of the recording chamber (APC-30, Asteck Co., Kasuya, Japan), which was maintained at 28.0°C. During recording, slices were placed on MEA substrates (MED-P515A, Alpha MED Scientific, Ibaraki, Japan) and covered with nylon mesh and a stainless slice anchor. An alternative way to couple a brain slice to an MEA substrate is to use cellulose nitrate as a coating pre-treatment (Egert et al., 2002).

Each of the 64 Chs in the array covered an area that was 50 × 50 μm^2^, and the distance between the centers of the adjacent sites was 300 μm. Each brain slice was carefully located on an MEA substrate, and the arrangement details between an electrode array and the brain slice including the auditory cortex were described in our previous report (Yamamura et al., 2017). The top electrode row was always located at cortical layer 1, so that layer 6 was always located in the fourth or fifth row from the top electrode row.

In each slice on the MEA probe, evoked LFP responses driven by ultrasound stimulation were recorded at a sampling rate of 20 kHz, and the signals were filtered using a range of frequencies between 100 Hz and 10 kHz. The locations of all recording positions on the multielectrode substrate were digitally imaged before and after recording.

Before conducting the main recordings, we recorded current-driven responses to examine the stability of the brain slices. We applied a bipolar square-pulse stimulus (intensity, 50 μA; duration, 200 μs) from one electrode located in layer 2/3 or layer 4: either one of Chs 11–14 (second row in the 64-ch array) or one of Chs 19–22 (third row). To monitor the excitability of brain slices in our main sessions, we performed alternating ultrasound or bipolar current square-pulse stimulation (the same as in the test session: intensity, 25 μA; duration, 200 μs) from one electrode located in layer 2/3 or layer 4 to the slice; one of Chs 11–14 and Chs 19–22. Each stimulation block consisted of 20 trials with the same stimulation conditions and a time interval of 5 s. The pair of blocks was repeated at least three times (total of 30 trials) with the same stimulation parameters. Overall, we obtained 351 ultrasound-evoked responses and 1,069 current-evoked responses for seven brain slices obtained from five animals. The number of current-evoked responses was around three times larger than the ultrasound-evoked responses because the current-evoked responses included those obtained from the test sessions prior to the main sessions, as well as post sessions conducted after the main sessions for the control experiments.

To distinguish ultrasound-driven responses from stimulation artifacts, tetrodotoxin (TTX), one inhibitor of voltage-gated sodium channels, was applied to the ACFS solution perfused in some experiments. The application of 1-μM TTX in the ACSF solution significantly reduced ultrasound-driven LFP responses or completely blocked the evoked activity (Supplementary Figure 1).

### Pattern Classification

To classify a current-driven LFP response pattern evoked at one stimulation site in one cortical layer (layer 2/3 or layer 4) as one of several clusters, we considered the individual pattern as a matrix ***A*** of 8 × 8 (64) elements. Peak amplitudes were recorded at each electrode site during the period 100 ms after the ultrasound stimulus onset and during the period from 3 to 40 ms after the current stimulation onset. Each matrix ***A*** was subsequently transformed into a corresponding vector (***a***) located in a multidimensional space. To achieve this, we first constructed matrix ***A*** for each response pattern, as follows. When an electrical stimulation was applied at an electrode site, for example, channel *p* (*p* = 1, …, 24, i.e., channel numbers from the top electrode row) in layer *q* (*q* = 2/3 or 4), the peak amplitudes of the LFP responses at 63 sites were detected as non-negative values, and the element value of the stimulation site was set to be zero. We denoted the elements of matrix ***A*** as *A_*m*,*n*_*, where subscripts *m* and *n* represent the row and column numbers of the electrode array, respectively, and are both integers (i.e., *m, n* = 1, …, 8). Then, we denoted a labeled response vector, ***a****_*p*_*, as a vector of all the elements of ***A*** with the label *p* (one stimulation channel). This response vector represents the specific layer and channel where the stimulation site is located. Similarly, the ultrasound-driven response pattern was represented as a matrix ***B*** with 8 × 8 (64) elements and the peak amplitudes of the LFP response, and the corresponding variable ***b*** was obtained as a vector of all the elements of ***B***.

After we obtained a spatiotemporal response pattern as the vector variable ***a****_*p*_* (or ***b***), we next applied a pattern classification method. In this study, we used a hierarchical clustering method (Duda et al., 2001). We constructed a set of unlabeled sample vectors from all of the vectors ***a****_*p*_* (or ***b***) obtained in our experiments. To perform clustering, we followed the following three steps (Duda et al., 2001). (i) To determine the similarity (dissimilarity) between each vector pair, the Euclidean distance was calculated. (ii) Vector pairs that were in close proximity were linked to each other, and a binary, hierarchical cluster tree (dendrogram) was constructed. (iii) Finally, a branch point was determined in the tree, so that all vectors below the branch point in the direction of singleton clusters were grouped into individually identical clusters. In this study, we classified the vectors ***a****_*p*_* and ***b*** obtained from the current-driven and ultrasound-driven responses into seven and five clusters, respectively. The centers of the clusters were determined *via* k-means clustering (Duda et al., 2001). As usual, the Euclidean distance (*d*_euc_) was defined as follows:


(6) $$d_{\text{euc}}\left(a,b\right)=\sqrt{\sum_{i=1}^{64}{\left(a_{i}-b_{i}\right)}^{2}}.$$

In this study, the similarity values (*I*_sim_) were defined as *I*_sim_ = (1 - *d*_norm_), where *d*_norm_ is the normalized value of *d*_euc_ with respect to the maximum for all distances.

In the initial state of clustering, we assumed two clusters and calculated the inter-cluster distance between the two geometric centers of the clusters. Then, while the number of clusters was increased, the inter-cluster distances were repetitively calculated for the corresponding cluster centers in all cluster combinations. If all inter-cluster distances were over a threshold value (0.5), the repetitive calculation was terminated, indicating that the distances between any two clusters were relatively further apart, compared with the initial state. Although the threshold value was determined ad hoc, shifting the threshold value ±0.1 did not profoundly change the terminated number of clusters. Additionally, to visualize the clusters consisting of individual response patterns, we computed a 3D subspace using principle component analysis (PCA) and constructed the corresponding vectors in the reduced dimensional space by projecting all original vectors onto the three principal components in the PCA space (Szymanski et al., 2011). As is usual for PCA, we calculated the contribution ratios, which represent the importance of each component in the data set. In our PCA calculation, the contribution of the first three principal component variables was more than 74%.

To characterize the similarity between any two response patterns, we calculated the correlation (*R*) of a pair of two centroid vectors ***c*_*a*_** and ***c*_*b*_** in the PCA space with the first three principal components. Here, the correlation *R* between two vectors was calculated as


(7) $$R\left({\text{c}}_{a},{\text{c}}_{b}\right)=\frac{\sum_{i=1}^{3}c_{ai}c_{bi}}{\sqrt{\sum_{i=1}^{3}c_{ai}^{2}}\cdot \sqrt{\sum_{i=1}^{3}c_{bi}^{2}}},$$

where *c*_*ai*_ and *c*_*bi*_ denote the *i* components of vector ***c*_*a*_** and ***c*_*b*_**, respectively.

Furthermore, we analyzed the pattern similarity between an ultrasound pressure distribution obtained from the numerical simulation and the LFP response peak values, under the condition that the centers of the transducer and the MEA recording area were out of alignment. To characterize the similarity between the ultrasound pressure distribution and the LFP response peak values, we constructed a matrix ***V*** (*x*_0_, *y*_0_) from the numerically simulated pressure distribution *I* on an MEA probe surface. The position of (*x*_0_, *y*_0_) represents the center of the recording area over the MEA probe, while the center of the acoustic pressure distribution is always located at the origin of the 2D space (i.e., *x* = 0 and *y* = 0 mm). When the center position of the recording area is at (*x*_0_, *y*_0_), the pressure distribution *I’* centered at that position was described as *I’*(*x, y*) = *I* (*x + x*_0_, *y + y*_0_), indicating just the shift in the position of the pressure distribution center. Because the centers of the recording electrodes are 0.30 mm apart, an element (*U*_*m,n*_) in the electrode pressure matrix ***U*** is defined as


(8) $$U_{m,n}\left(x_{0},y_{0}\right)=I^{\prime }\left(m△x-1.05,n△y-1.05\right)=I\left(m△x-1.05+x_{0},n△y-1.05+y_{0}\right),$$

where Δ*x* and Δ*y* are inter-electrode intervals (i.e., Δ*x* = Δ*y* = 0.30 mm), and *m* and *n* are row and column numbers (both integers, i.e., *m, n* = 1, …, 8). After normalizing the maximum value of the matrices ***U*** (*x*_0_, *y*_0_), we finally obtained the normalized matrix ***V*** (*x*_0_, *y*_0_).

### Data Analysis

The data are based on experiments conducted in seven brain slices that included the auditory cortex from five C57BL/6J mice. Data are given as the mean ± standard error of the mean (SEM). Statistical analysis for comparisons involving multiple groups was performed using an ANOVA for multiple comparisons followed by a *post-hoc* Tukey–Kramer test. *P* < 0.05 was taken to be statistically significant. For template matching, we used the Python-wrapped image analysis library (OpenCV).

## Results

### Simulation of the 2D Model and Determination of Waveguide Structure

We first explored the size parameters (*d* and *l* in Figure 1Aa) of the waveguide structure to ensure that it satisfied the conditions of the peak intensity (≥10.0 W/cm^2^) and half width (≤2.1 mm) on the surface of an MEA probe driven by ultrasound stimulation to the backside of the MEA probe. The waveform of the transducer pressure was sinusoidal, with an amplitude of 100 kPa. The typical pressure distribution for the cross section of the waveguide and the MEA chamber is shown in Figure 2A. For a fixed waveguide size of *l* (*l* = 15, 20, or 25 mm), the relationships between peak intensity (*I*_sppa_ of Eq. 5) and half width as a function of the diameter (*d*) of the waveguide mouth are illustrated in Figures 2Ba,b, respectively.

When the diameter *d* of the waveguide mouth was increased, the peak intensity first increased and was almost saturated over 4 mm (Figure 2Ba). Similarly, when the diameter was increased, the half width was increased (Figure 2Bb). The amount of ACSF solution in the MEA chamber was varied with ten height levels (*h*) ranging from 1.0 to 10 mm in 1.0-mm steps; the error bars in the two plots show the standard deviations.

Among the several candidates for the two size parameters (*d* and *l*), we finally selected *d* = 4 mm and *l* = 20 mm as the 3D model parameters. These satisfied the two conditions: the peak intensity was 10.1 W/cm, and the half width was 2.03 mm. Furthermore, under the same size parameter conditions, the focal length [peak position in the vertical (z) axis] was 0.9 mm from the bottom of the MEA probe (0.7 mm thickness), which indicates that the focal point was located within the brain slice (0.4 mm thickness).

### Simulation of the 3D Model and Pressure Patterns in a Brain Slice

**FIGURE 4:**
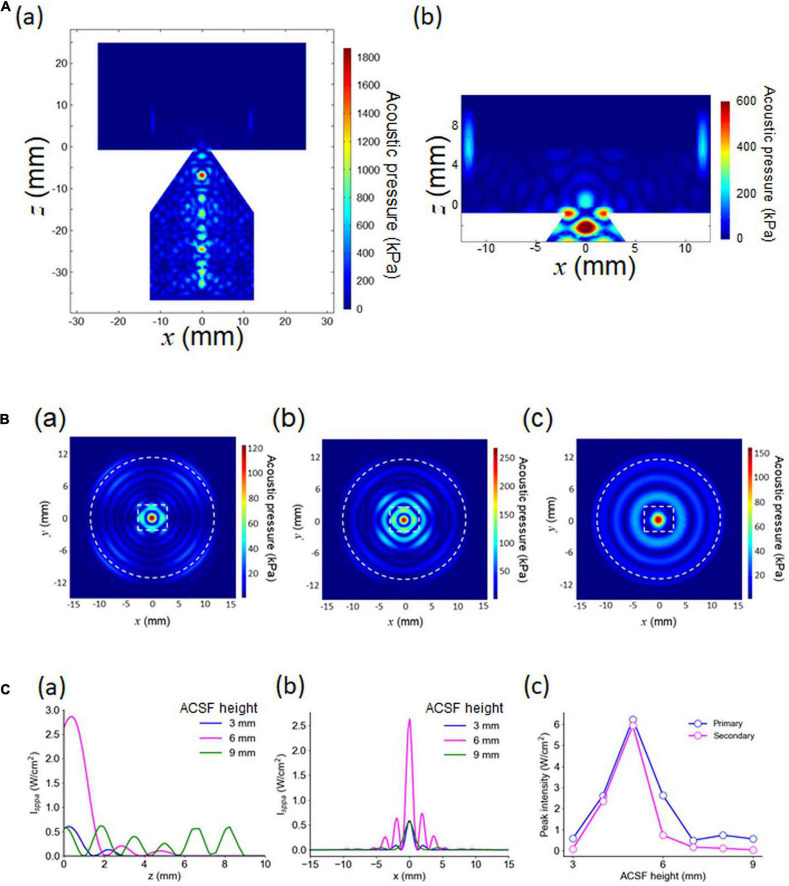
Numerical results of 3D modeling. **(A)** In (a), the acoustic pressure distribution in the vertical cross section passing through the central axis. The size parameters are *d* = 4.0 mm and *l* = 20 mm. In (b), an expanded view of (a), including a cross-section of the MEA chamber, for the same size parameters. **(B)** The acoustic pressure distribution in the horizontal cross section located at *z* = 0 mm in (a) (height = 35.7 mm in Figure 2A). The height of the ACSF solution in the chamber was the only difference among the three plots; the other parameters were exactly the same. The three plots in (a–c) illustrate the pressure distribution patterns for a fluid height of 3, 6, and 9 mm, respectively. **(C)** Intensity (*I*_sppa_) distributions for vertical and horizontal cross sections. In (a), the intensity distribution passing through the central axis (*z* ≥ 0; *x* = 0 and *y* = 0 in mm) is shown for a fluid height of 3, 6, and 9 mm. In (b), the intensity distribution on the horizontal plane at *z* = 0 with *y* = 0 in mm. In (c), a summary of the peak intensity, numerically obtained as a function of the height of the ACSF solution in the MEA chamber.

To examine pressure distribution patterns in a brain slice, we simulated the corresponding 3D model (Figures 3A,C) using the FEM. In the 3D model, a rectangular brain slice of 4.0 × 3.5 × 0.4 mm^3^ was assumed to be located in the center of the MEA recording area (Figures 1Ab, 3A, 4B). As a waveguide model, the size parameters of *d* = 4 mm and *l* = 20 mm were used, which was determined on the basis of the 2D model simulation. The vertical cross section of the pressure distribution in response to sinusoidal ultrasound stimulation (100 kPa) is illustrated in Figure 4A; the top height (*h*) of the ACSF solution in the MEA chamber was 6 mm. The result obtained from 3D modeling (Figure 4A) was similar to that obtained from 2D modeling (Figure 2Aa), although the colormap plots appear different at first glance.

The horizontal cross sections of the pressure distribution at *z* = 0 mm (the bottom plane of a brain slice) in Figure 4Aa are illustrated in Figure 4B. For waveguides that are the same size (*d* = 4 mm and *l* = 20 mm) with different amounts of ACSF solution in the MEA chamber (*h* = 3, 6, and 9 mm), three typical examples of pressure distribution patterns for a brain slice are shown in Figure 4B. Figure 4Bb (*h* = 6 mm) shows several decaying local peaks along the radial axis, with an approximately 2-mm interval on the MEA chamber. This result suggested that concentric standing waves induced by the ultrasound stimulation would be observed around the center of the MED chamber in the actual pressure measurement experiment.

We found that the pressure distributions details depended on the height of the ACSF solution in the chamber (Figures 4B,C). For example, the amount of ACSF solution increased with the number of local peaks around the central peak (Figure 4C). Therefore, the spatial modes (local peak intervals) of acoustic pressure distributions may differ according to the height of the ACSF solution in the MEA chamber. In addition, the relationship between the intensity of the peaks (the largest and second largest peaks) and the height of the ACSF solution was non-monotonic (Figure 4Cc), indicating that this relationship could have a greater impact on the complexity of ultrasound-induced neural activity than the changes in ACSF.

### Pressure Measurement in the MED Chamber Using a Hydrophone

**FIGURE 5:**
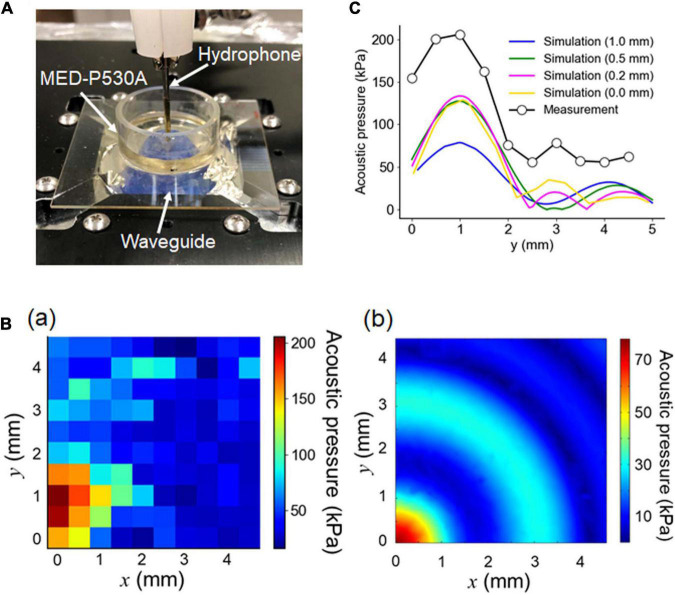
Pressure measurement using a hydrophone. **(A)** Actual image of the experimental pressure measurement setup. For the optimally designed waveguide attached to an ultrasound transducer, the fluid pressure in the MEA chamber was measured using a needle hydrophone located 1.0 mm away from the surface of the MEA substrate. The height of the ACSF solution was 6.0 mm. **(B)** For one quadrant of the circular chamber, the fluid pressure was spatially measured, as illustrated in (a). During the experiment, the hydrophone was repositioned in 0.5 mm increments to a maximum distance of 4.5 mm on each axis (*x*- and *y*- axes) from the center. The results of the corresponding numerical simulation for the 3D model, conducted under the same conditions, are illustrated in (b). **(C)** For the area of interest, the experimental pressure distribution on the *y*-axis (circle) and the corresponding simulated pressure distributions for the different distances (0, 0.2, 0.5, and 1.0 mm) between the MEA-substrate surface and the hydrophone tip were superimposed.

To examine the spatial irradiation properties of ultrasound waves traveling through the custom-designed waveguide attached to an ultrasound transducer, we measured the fluid pressure in the MEA chamber using a needle hydrophone located 1.0 mm from the surface of the MEA. The hydrophone was vertically fixed to the MEA probe in the chamber (Figure 5A). During the measurement, the height of the ACSF solution in the chamber was 3.0 mm.

For one quadrant of the circular chamber, the measured pressure is spatially shown in Figure 5Ba. We compared the measurement with the simulation using the simulated pressure in the corresponding area under the same conditions, as shown in Figure 5Bb. Although the radial symmetry of the pressure distribution was clear in the numerical simulation, the symmetry was not evident in the measurement. While the measurement (in mm) traveled from the origin (0, 0) to (4.5, 4.5) for the sensing area, the time lags among the individual measurements on the 2D plane might have influenced the pressure sensitivity. The difference may have been caused by dewatering between the top of the waveguide and the glass MEA substrate, which was conducted to reduce the impedance mismatch between them.

The measured and simulated pressures in the radial axis are superimposed in Figure 5C. In the simulation, the sensor (hydrophone) position was varied with respect to the MEA substrate from 0 to 1.0 mm. Figure 5C indicates that, under the condition in which the sensor position was 1.0 mm above the surface of the MEA substrate, the second largest peak in the measured pressure (black dot) was around 2 mm away from the largest peak. In contrast, under the same conditions, the second largest peak in the simulated pressure (blue curve) was around 3 mm away from the corresponding largest peak. In the simulation, the second peak positions shifted toward the largest peak position as the distance between the sensor and the MEA substrate decreased: e.g., 0.5, 0.2, and 0 mm. This implies that the measurement distance between the sensor and MEA substrate might be smaller than 1.0 mm, although the measured pressures were larger than the simulated pressures. In the pressure measurement, the use of a phantom slice (or brain tissue block) may give a more accurate result consistent of the numerical simulation and an appropriate representation for the biological experiment.

### Spatiotemporal Properties of Current-Driven Local Field Potentials

**FIGURE 6:**
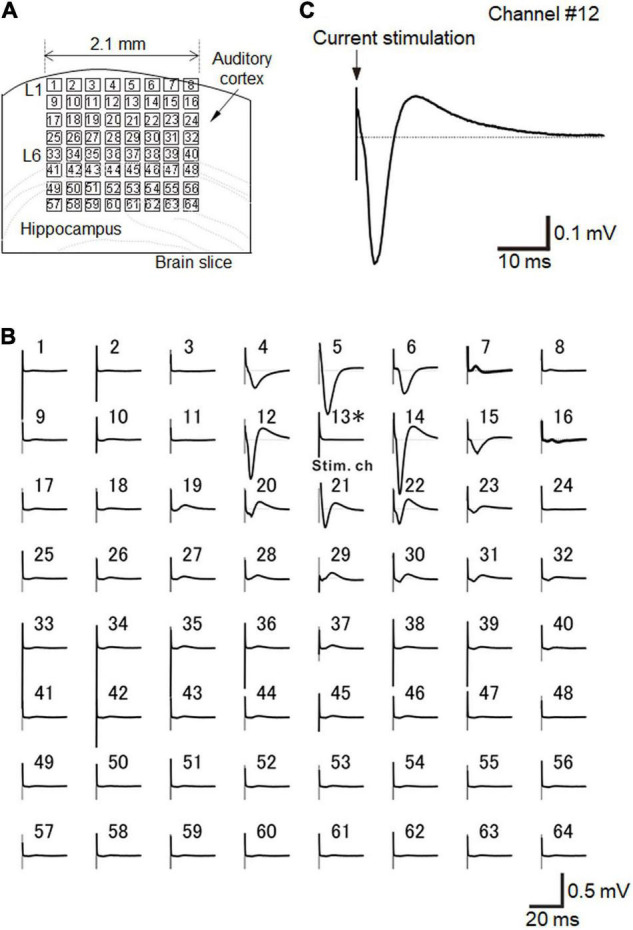
Brain slice arrangement and current-evoked responses. **(A)** Schematic diagram of a cortical coronal slice on an MEA substrate. Small squares represent the 64 electrodes. The size of each electrode was 50 × 50 μm^2^, and each inter-electrode interval was 300 μm. **(B)** In a slice of the mouse neocortex that included the auditory cortex, evoked LFPs were extracellularly recorded on the MEA substrate with 64-channel (Ch) electrodes. Averaged LFP responses evoked *via* current stimulation at a single electrode (Ch 13), which was in layer 2/3. Each average waveform was obtained over 10 trials. The stimulation current intensity was 25 μA. **(C)** Typical LFP response waveform recorded at Ch 12 in response to a short current stimulation. The LFP response trace is the same as in **(B)**. Similarly, the average waveform was obtained over 10 trials.

Before and after the ultrasound stimulation experiment, we recorded the activity of brain slices *via* electric current stimulation, as described in the experimental protocol. As a typical example, when a short current pulse at a low intensity (25 μA) was applied to a brain slice that included the auditory cortex on the MEA probe in a fixed arrangement (Figure 6A), we obtained highly reproducible extracellular LFPs (Figure 6B).

For the recordings, we selected a single stimulation electrode (e.g., Ch 13 in Figure 6A), and the electrode site was located at layer 2/3 or layer 4 (Figure 6A). In layer 2/3, typical LFPs in auditory cortex slices during a 50-ms time window after stimulation onset had three components: (i) an early negative-going potential (component A) from the stimulation onset to approximately 3 ms afterward; (ii) a late positive-going potential (component B) following component A; and (iii) a final decaying potential that approached the initial baseline (component C) (Figure 6C; Yamamura et al., 2017). If the stimulation channel was at layer 5/6, the evoked responses propagated into the upper layers (data not shown; Yamamura et al., 2017). Consequently, the response amplitudes were maximized in layer 2/3 (Yamamura et al., 2017). In this study, we only selected single channels at layer layer 2/3 or layer 4 for each stimulation trial.

**FIGURE 7:**
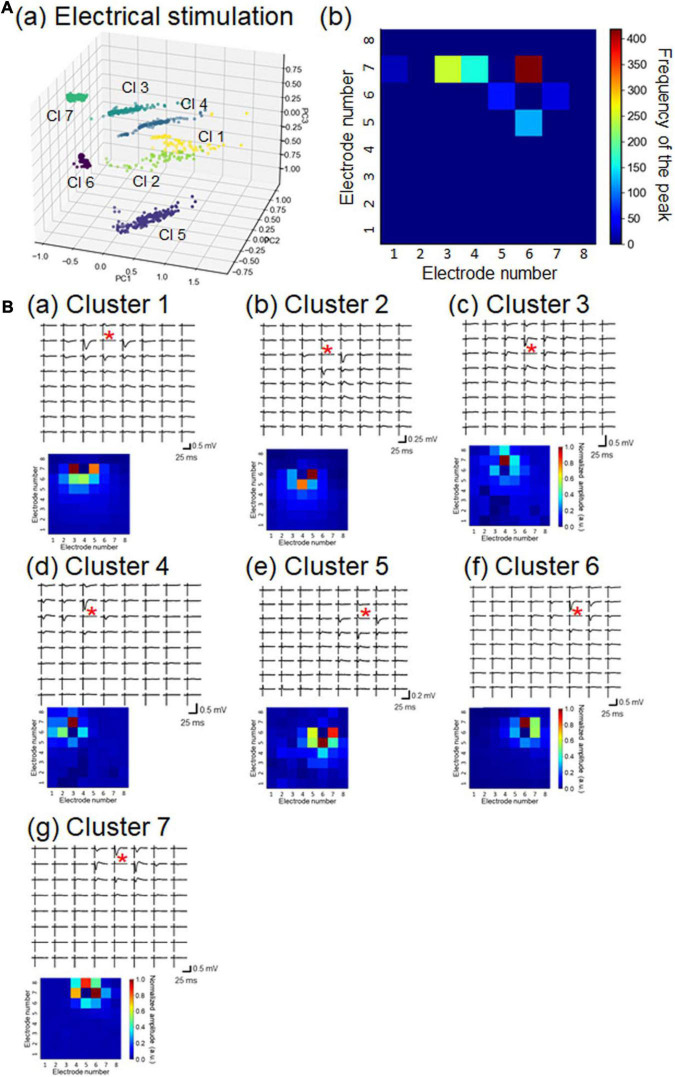
Properties of LFP response patterns for current stimulation and pattern classification. **(A)** A total of 1,069 LFP patterns were converted into pattern vectors **a**_*p*_. The vectors were plotted in 3D space for three major components, calculated using standard principal component analysis (PCA), shown in (a). The vectors are classified into seven clusters (Cls): Cls 1 to 7. In (b), for each response pattern, the number of sites for which the maximum amplitude was at a negative peak was counted and accumulated on a 2D electrode array. The colormap shows the frequency of the maximum amplitude sites among the 64 electrode sites. **(B)** For each cluster, a typical LFP response (upper) and associated pattern matrix (lower) with elements that represent the peak amplitudes. Each stimulation site is indicated by a red asterisk in each LFP response plot.

To characterize the 1,069 LFP patterns obtained in response to current stimulation, we converted them to pattern vectors ***a****_*p*_* and classified the vectors into seven clusters (Figure 7Aa). All inter-cluster distances were over the pre-set threshold value (>0.5), indicating that the distances between any two patterns belonging to different clusters were relatively far apart. Moreover, because we selected stimulation sites at layer 2/3 or layer 4, the maximum amplitude responses were usually obtained from the recording sites in the second, third, or fourth row in the MEA array (Figure 7Ab). A typical LFP response and the pattern matrix ***A*** from each cluster are illustrated in Figure 7B. In particular, pattern pairs between clusters 1 and 2 (1, 2), 3 and 4 (3, 4), and 6 and 7 (6, 7) had larger similarity index values (i.e., smaller Euclidean distances). All of these results, including the number of patterns in each cluster, are summarized in Supplementary Table 1.

In short, the obtained response patterns in individual clusters reflected the corresponding stimulation sites and the propagation properties of current-evoked activity in local cortical networks. Identical stimulation sites did not always induce the same response patterns, implying that the propagation properties of individual brain slices varied to some extent.

### Spatiotemporal Properties of Ultrasound-Driven Local Field Potentials

**FIGURE 8:**
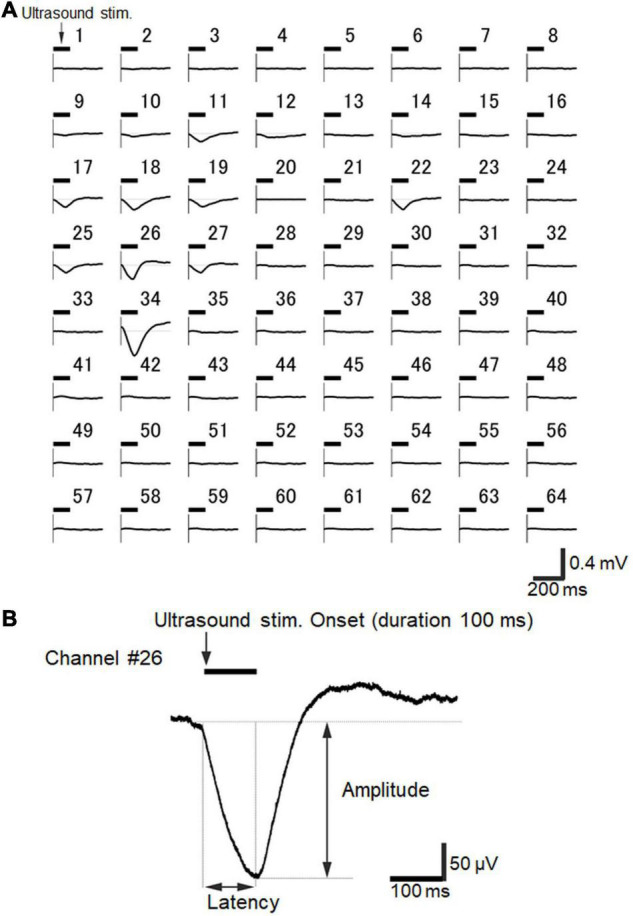
Ultrasound-driven LFP responses recorded over the 64 electrodes of the MEA probe. **(A)** A typical example of spatiotemporal ultrasound-driven responses, where the ultrasound stimulation was delivered to the same slice shown in Figure 6. The stimulation pressure of the transducer was 210 kPa, and the stimulation duration was 100 ms. The timing of the ultrasound stimulation is illustrated by a horizontal bar on the upper left side of each plot of individual LFP responses for recorded electrodes. Each average waveform was obtained over 5 trials. **(B)** Typical LFP response waveform recorded at Ch 26 in response to the same ultrasound stimulation. The LFP response trace is the same as shown in A. The average waveform was obtained over 10 trials. Response amplitude and latency are also indicated in the plot.

Next, we examined whether ultrasound stimulation could actually evoke neural activity in a brain slice when our MEA-based recording system was combined with the custom designed waveguide. Ultrasound-driven LFP responses were recorded among seven examined slices. See Figure 8A for examples of typical spatiotemporal ultrasound-driven responses for an ultrasound stimulation with a 100-ms duration, delivered to the same slice shown in Figure 6. For this brain slice, larger responses were locally observed in layer 5/6, although cortical layer-specificity of evoked responses was not always easily characterized. This example demonstrates that the responses were not only obtained from a small local area (e.g., Chs 26, 27, and 34), but from several response subareas (Chs 14 and 22) as well. In addition, with respect to the time courses of LFPs, responses with a very slow transient component were obtained from several electrodes after the ultrasound stimulation onset (Figure 8A). The time scale for typical ultrasound-driven responses was approximately 10 times larger than that for current-driven responses (i.e., Figure 6 vs. Figure 8). As an exception, faster LFP responses were obtained from one brain slice (see below). Furthermore, for ultrasound stimulation with a 100-ms duration, typical LFPs in auditory cortex slices evoked during the 500-ms time window after stimulation onset were also composed of three components (Figure 8B): (i) an early negative-going potential (component A) from the stimulation onset to approximately 100 ms afterward; (ii) a late positive-going potential (component B) following component A; and (iii) a final decaying potential (component C) approaching the baseline level. Here, we characterized the LFP waveforms using two response properties: peak latency and peak amplitude (Figure 8B).

**FIGURE 9:**
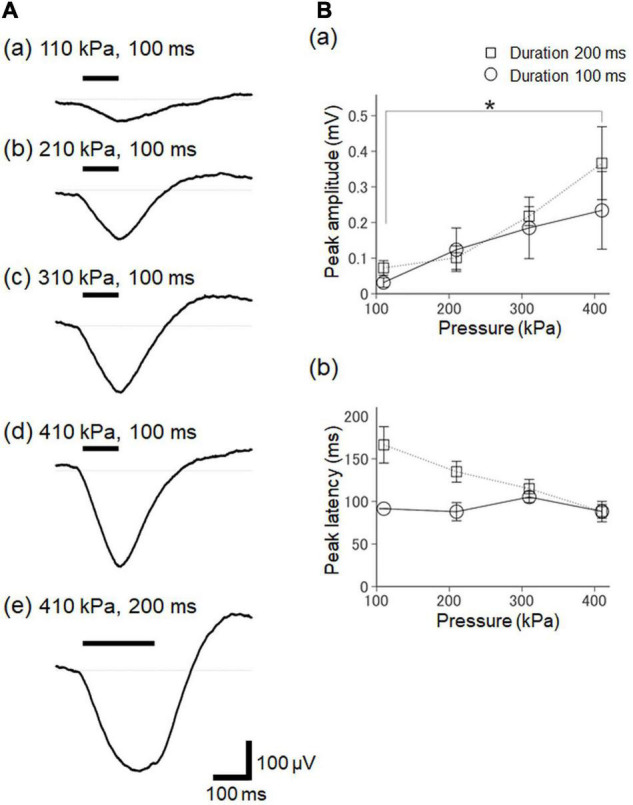
The dependency of ultrasound-driven LFP responses on stimulation intensity and duration. **(A)** A typical example of elicited LFPs for various pressure and duration stimulation parameters. The values of the stimulation parameters are described in the plots. The timings of the ultrasound stimulation are illustrated by a horizontal bar on the upper left. **(B)** Summary of ultrasound-driven LFP response properties for different ultrasound pressures in two groups with the different durations (100 and 200 ms) of ultrasound stimulation. In (a), the relationship between peak amplitudes and stimulation pressure is illustrated for seven brain slices. In (b), similarly, the relationship between peak amplitudes and stimulation pressure is illustrated for five slices. We used the Tukey-Kramer test for multiple comparisons, * represents *P* < 0.05.

The peak amplitudes increased with the acoustic pressure levels of the ultrasound stimulation (Figures 9Aa–d) for the two stimulation durations (100 and 200 ms; Figure 9A). In response to the ultrasound stimulation with a 200-ms stimulus duration, the differences in the peak amplitude of the ultrasound-driven LFP responses were significant between the smallest pressure (110 kPa) and the largest pressure (410 kPa) levels (paired *t*-test, *P* = 0.042, Figure 9Ba), indicating that the pressure level of the ultrasound substantially impacted the LFP response. In addition, the pressure levels of the ultrasound stimulation with a 100-ms duration did not influence the peak latencies in the early phase of the evoked response, and the latencies were around 100 ms (Figure 9Bb). In contrast, as the pressure levels of the ultrasound increased with the 200-ms stimulation duration, the latencies of the large negative peak shifted to around 100 ms (Figure 9Bb). This result also confirmed that the pressure levels of the ultrasound stimulation closely reflected the LFP responses, and that they more strongly influenced the peak amplitudes than the peak latencies. Furthermore, the duration of the ultrasound stimulation (100 vs. 200 ms) influenced the duration of the LFP response, and the response durations were such that the temporal lengths of the LFP response amplitude >50% of the corresponding peak amplitude. For the pressure of 310 kPa, for example, in response to the ultrasound stimulation with the durations of 100 ms and 200 ms, the LFP response durations were 69.0 ± 20.4 ms and 128.0 ± 33.5 ms, respectively. This result indicates that the LFP response durations, which were significantly different, were dependent on the ultrasound durations (paired *t*-test, *P* = 0.028). Therefore, for the examined ultrasound range, the temporal integration effect of the ultrasound stimulation was proportional to the stimulation duration.

**FIGURE 10:**
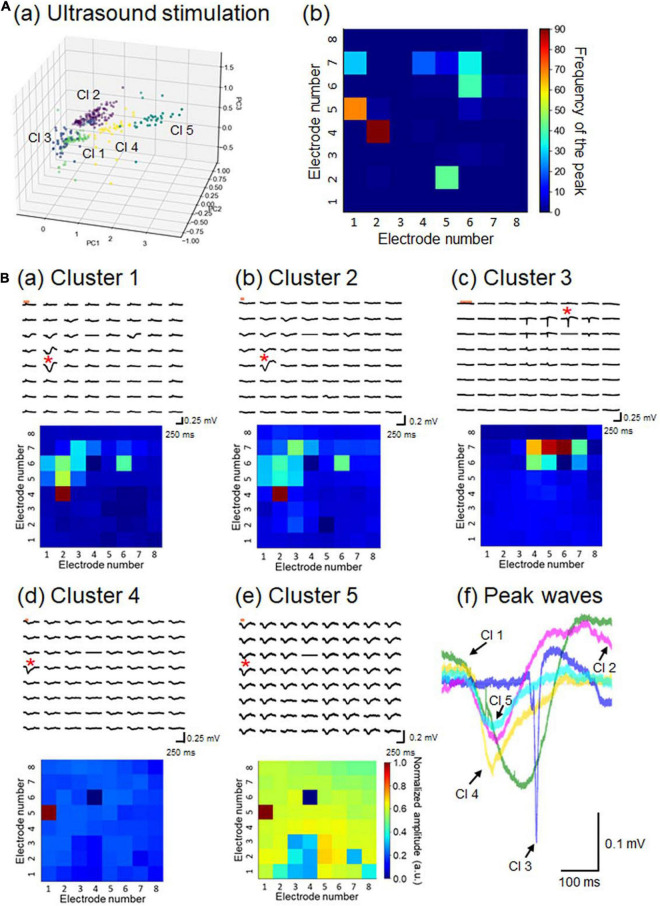
Properties of LFP response patterns for ultrasound stimulation and pattern classification. **(A)** A total of 351 LFP patterns were converted into pattern vectors **a**_*p*_. These vectors were plotted in 3D space for three major components, calculated using the standard PCA in (a). The vectors were classified into five clusters, labeled Cl 1 to Cl 5. In (b), for each response pattern, the number of sites with a maximum amplitude at a negative peak was counted and accumulated on a 2D electrode array. The colormap represents the frequency of the maximum amplitude sites among the 64 electrode sites. **(B)** For each cluster, a typical LFP response (upper) and its corresponding pattern matrix (lower), with elements representing the peak amplitudes, are illustrated in (a) to (e), as in Figure 7B. In (f), for a typical response pattern for each cluster, an LFP response waveform with the maximum amplitude is superimposed for all 5 clusters. The selected electrode sites are indicated by red asterisks in the LFP response plots.

To characterize the 351 LFP patterns obtained in response to the ultrasound stimulation, we converted them to pattern vectors ***b*** and classified the vectors into five clusters (Figure 10Aa). All inter-cluster distances were over the pre-set threshold value (>0.5), although some clusters seemed to be close to one another (e.g., clusters 1 and 2). For each response pattern, the number of sites for which the maximum amplitude was on a negative peak was counted and accumulated on a 2D electrode array, and the results indicated that the sites of the peaks driven by ultrasound stimulation were more dispersed (Figure 10Ab) than those with current stimulation (Figure 7Ab). A typical LFP response and a pattern matrix ***B*** from each cluster are given in Figure 10B. In cluster 3, the time courses of the LFP responses obtained from one brain slice were faster than the response time-courses from the other clusters, and the response latencies were longer after the stimulation onset (Figure 10Bf). In cluster 5, negative-going responses were obtained for almost all sites, implying that the ultrasound stimulation was effective for evoking neural activity. Therefore, the obtained response patterns in the individual clusters appeared to reflect the corresponding ultrasound pressure distributions and propagation properties of ultrasound-evoked activity in particular local networks. All of these results, including the number of patterns in each cluster, are summarized in Supplementary Table 2.

**FIGURE 11:**
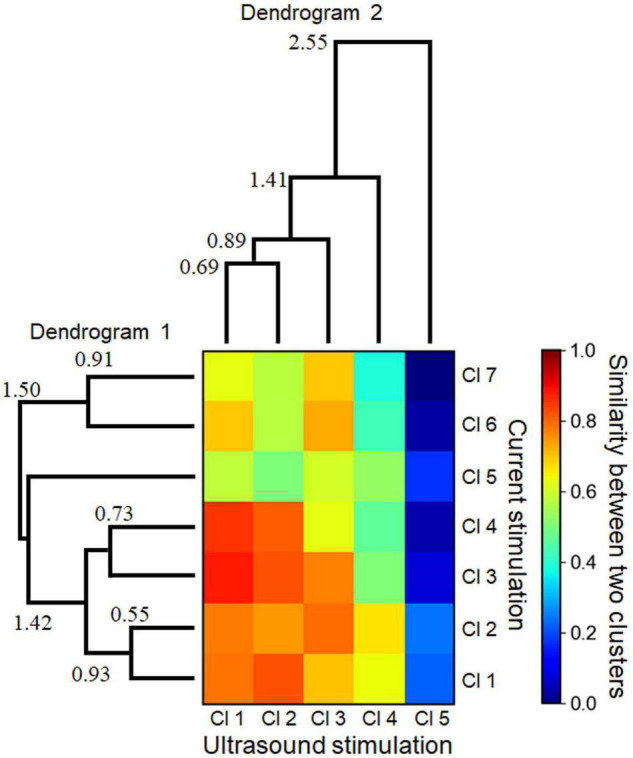
Similarities between two cluster sets with respect to the response patterns driven by electrical and ultrasound stimulations. A heatmap shows the normalized similarity between the two cluster sets. To calculate the similarity of the values in the heat map, Euclidean distances between the centroids of the individual clusters were calculated, and the similarity values were found to be inversely proportional to the distances. Two dendrograms (linkage tree diagrams) illustrate the cluster sets for the electrical stimulation (dendrogram 1) and ultrasound stimulation (dendrogram 2). The numbers in the dendrograms represent the corresponding distance values between the linked clusters. The minimum values (0.55 and 0.69 in dendrograms 1 and 2, respectively) were over the preset threshold (>0.5).

The correlation coefficients between two response clusters obtained from current- and ultrasound-driven stimulations are illustrated in Figure 11. We created dendrograms of the clusters obtained from the individual stimulation methods by characterizing the similarities among the cluster pairs for each stimulation method (Figure 11). Although the response times differed in size by approximately 10-fold between the two stimulation methods, some clusters had similar response properties in the 2D space across the two stimulation methods. In particular, three cluster patterns (clusters 1–3 in both stimulations) were closely matched (similarity index ≥ 0.794 in Figure 11). However, the patterns in cluster 5 showed dissimilarity (similarity index < 0.3) with all response patterns obtained by current stimulation.

### Analyzing the Similarity of Response Patterns Using Ultrasound Pressure Distributions

**FIGURE 12:**
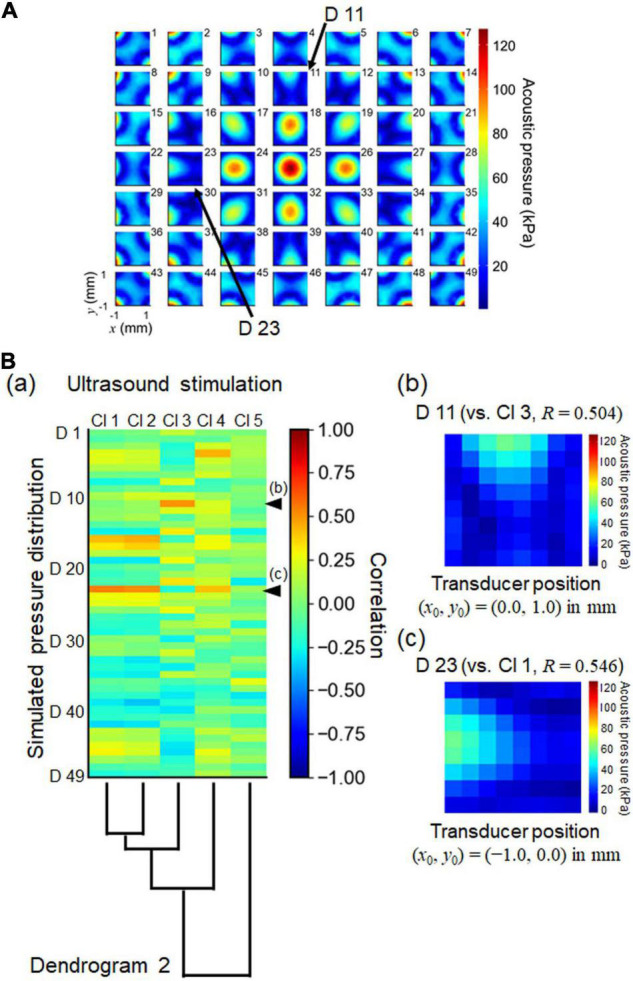
Numerically simulated patterns of ultrasound pressure distributions and normalized cross correlation with the cluster set obtained by ultrasound stimulation. **(A)** The illustration shows 49 (7 × 7) patterns of numerically simulated pressure distributions. Each pattern was calculated from a pressure distribution in which the center position (*x*_0_, *y*_0_) of the ultrasound transducer was simply shifted by a step of 0.5 mm relative to the center of the MEA substrate. For the pattern labeled “25,” the center position was located in the origin of the MEA substrate: (*x*_0_, *y*_0_) = (0, 0). **(B)** In (a), the heatmap represents the correlation coefficients between the simulated pressure patterns and the centroids of the clusters associated with the responses driven by ultrasound stimulation. Dendrogram 2 from Figure 11 is plotted again. In (b) and (c), two simulated pressure patterns (D11 and D23, respectively) are illustrated, as indicated by arrows in **(A)** and arrow heads in **(B)** (a). The correlation coefficients (*R*) of D23 vs. Cl2 and D11 vs. Cl3 are 0.544 and 0.622, respectively. The center positions of the transducer also are shown in (b) and (c) as (*x*_0_, *y*_0_) = (0, + 1.0) and (*x*_0_, *y*_0_) = (−1.0, 0) in mm, respectively.

During the experiments, we were careful to ensure that the center of the transducer coincided with the center of the recording area on the MEA substrate. However, the pressure distribution measurements suggested that the center of the pressure distribution did not always match the exact center of the MEA substrate (Figure 5Ba). Therefore, we examined how discrepancies in the center position could affect ultrasound pressure distributions and pattern similarities. Here, we assumed that the one-site neural response driven by ultrasound stimulation would be proportional to the ultrasound pressure distribution at the corresponding electrode site. In our numerical calculation, we artificially simulated 49 (7 × 7) pressure distribution patterns, in which the center position (*x*_0_, *y*_0_) of the ultrasound transducer was simply shifted by a step of 0.5 mm relative to the center of the MEA substrate (Figure 12A). We found that ultrasound-driven clusters 1, 2, and 3 were most similar to the pressure distribution patterns (D 23 and D 11 in Figure 12A). Their transducer centers were 1.0 mm leftward [i.e., transducer-center position, (*x*_0_, *y*_0_) = (−1.0, 0) in mm] and 1.0 mm upward [(*x*_0_, *y*_0_) = (0, +1.0) in mm]. The correlation coefficients (*R*) of D 23 vs. Cl 1 and D 11 vs. Cl 3 were 0.546 and 0.504, respectively. The correlation coefficients between all of the ultrasound clusters (Cl 1 to Cl 5) and all artificial pressure distributions (D 1 to D 49) are given in Figure 12Ba. These results of the numerical calculation and the correlation analysis indicate that the alignment between the centers may influence the spatial patterns of ultrasound-driven neural responses. Therefore, a measurement system that is capable of fine alignment is desirable for future research.

## Discussion

In this study, we constructed an MEA-based recording system combined with an ultrasound transducer and a waveguide to record activity driven by an ultrasound short burst oscillation in a brain slice. First, to determine the appropriate irradiation properties for stimulating a brain slice *in vitro*, we performed a computer simulation of our experimental setup and designed a waveguide to be attached to the tip of an ultrasound transducer. We manipulated the size of the waveguide according to two conditions: peak intensity ≥10.0 W/cm^2^ and half width ≤2.1 mm on the surface of the MEA probe. Then, we produced the designed waveguide structure using a 3D printer and recorded extracellular activity in brain slices driven by our ultrasound stimulation setup. Finally, we analyzed the recorded neural activity driven by the ultrasound stimulation, and compared it with the response patterns from current-driven stimulation and simulated pressure distributions within the slice. We were able to use our *in vitro* experimental setup to directly examine the possibility of locally activating neurons in a brain slice *via* ultrasound stimulation. Our electrophysiological experiments indicate that ultrasound-evoked cortical responses in mouse brain slices depend on the intensity and duration of the ultrasound stimulation. Thus, our results show that choosing the appropriate ultrasound waveguide structure and stimulation parameters is important for producing the desired intensity distribution in a localized area within a brain slice.

### Selection of Pressure Pattern and Intensity for Ultrasound Stimulation

A variety of temporal patterns have been used for ultrasound stimulation of the brain (Rinaldi et al., 1991; Bachtold et al., 1998; Khraiche et al., 2008; Tyler et al., 2008; Choi et al., 2013; Menz et al., 2013; Kim et al., 2017; Kubanek et al., 2018; Prieto et al., 2018; Menz et al., 2019; Supplementary Table 3). In this study, we selected a simple sinusoidal oscillation (frequency, 500 kHz; duration, 100 or 200 ms) as the stimulation waveform. Many indices of intensity have been used to characterize the multiple time scales of ultrasound stimulation patterns (Supplementary Table 3). Here, we used the spatial-peak pulse-averaged intensity (*I*_sppa_) as one such index, simply defined as the average intensity of a sinusoidal wave pulse.

Repetitive bursts of short ultrasound oscillation pulses are often selected as another common stimulation pattern (Tyler et al., 2008; Choi et al., 2013; Kim et al., 2017). For these, the spatial-peak temporal-averaged intensity (*I*_spta_) is commonly used as an index, defined as the average intensity over the total duration of each stimulus. Burst parameters, including the burst duration and interval used in *I*_spta_, are not relevant to the present study. However, the effect of repetitive ultrasound bursts on neural activity in brain slices is of interest for future work because these bursts elicit stronger neural activation (Bystritsky et al., 2011; Blackmore et al., 2019). The stimulation intensities and carrier frequencies used in previous *in vitro* preparations are summarized in Supplementary Table 3.

To the best of our knowledge, this study is the first to report ultrasound stimulation-evoked responses in acute cortical slices from mice. In terms of diagnostic ultrasound imaging devices for human applications, the Food and Drug Administration (FDA) guidelines provide the following safety criterion regarding stimulation intensities: the *I*_spta_ must not exceed 720 mW/cm^2^ and the *I*_sppa_ must not exceed 190 W/cm^2^ (Duck, 2007). These intensities are much larger than those used in our experiments (*I*_sppa_ ≤ 5.68 mW/cm^2^), although *in vitro* and *in vivo* situations may vary in terms of acoustically reflective surfaces and the surrounding environment (i.e., *in vivo* vs. glass MEA probe, recording chamber, and brain slices in experimental ACSF solution). Understanding the limitations associated with *in vitro* systems is important when interpreting our experimental data and considering how our findings will be applicable to future work. In particular, the standing ultrasound waves that we detected in the MEA chamber could cause complex distributions of ultrasound intensity within slices, and this could reflect the observed response activity (e.g., Figures 4B,C).

### Possible Mechanisms for Ultrasound-Evoked Activity in Brain Slices

Although neural impulses have historically been considered to be electrical signals, the super-threshold depolarization mechanisms of neural membranes involve a combination of electrical, mechanical, chemical, and conformational changes in the excited cells. Thus, describing a neural impulse *via* a mechanical pathway could support a physical basis for ultrasound-driven neuromodulation. Several recent review papers (Blackmore et al., 2019; Pasquinelli et al., 2019; Cardenas-Rojas et al., 2020) summarized four potential mechanisms by which ultrasound signals could trigger action potentials: (i) the generation of capacitive currents as a result of membrane displacements, (ii) the activation of mechanosensitive channels, (iii) sonoporation in the lipid bilayer, and (iv) coupling with membrane waves along the axon. Effective ultrasonic neurostimulation may be possible by combining these mechanisms. In particular, to enable sufficient radiation force for larger ultrasound amplitudes, the conformational change could directly polarize the membrane potentials *via* mechanocapacitive coupling (Zecchi et al., 2017), resulting in rapid excitation without a time delay. In contrast, at lower ultrasound amplitudes such as those used in this study, the radiation force is weak, which may depolarize the membrane in the presence of very slowly depleting ion gradients. Therefore, the duration of a spike will need to be sufficiently long for polarization to cross the threshold required to produce an action potential.

### Ultrasound-Driven Local Field Potential Response Patterns and Pattern Classification

As reported in our previous studies (Namikawa et al., 2017; Yamamura et al., 2017), we reliably observed cortical layer-specificity of evoked responses among brain slices that included the mouse auditory cortex. In this study, we recorded spatiotemporal LFP patterns in response to current stimulation *via* a single electrode at layer 2/3 or layer 4 of mouse auditory cortical slices. A short current-pulse activated LFP responses around the stimulation site, and the activity propagations were locally restricted (Figure 6). In addition, the LFP patterns could be classified into seven clusters (Figure 7A) using a pattern classification method known as hierarchical clustering. Thus, the clustered patterns were closely dependent on the stimulation sites (Figure 7B) and the laminar properties of the cortical slices.

We recorded and analyzed activity patterns for MEA sites at which ultrasound-driven LFP responses were evoked (Figure 8), and found that the activity patterns could be classified into five clusters (Figure 10A). That ultrasound-driven LFP responses were more complex than the response patterns for current stimulation (Figure 10B vs. Figure 7B), implying that the ultrasound pressure distribution itself was complex (e.g., Figure 4B). Furthermore, among the two response cluster sets, we compared the response patterns driven by current and ultrasound stimulations. We found that three cluster patterns (clusters 1–3 in both stimulations) were closely matched (similarity index ≥ 0.794 in Figure 11). In addition, we compared the response patterns with the patterns of ultrasound pressure distributions, and found that some pressure patterns were matched with ultrasound-driven response patterns (Figure 12). Thus, the response similarity appears to be influenced by the laminar properties of cortical slices and/or spatial patterns of ultrasound stimulations. We plan to investigate this experimentally in our future work.

### Improving the Present Measuring System

One factor over which we had limited control was the volume of the ACSF solution flowing in the MEA chamber. We are planning to construct a feedback control system to regulate this in our future work. Using such a control system, we expect to find that ultrasound-driven cortical responses have more similar activity patterns among different cortical slices, reflecting the relationship between ultrasound pressure patterns and neural response patterns.

In addition, in our future MEA-based recording system, we hope to improve the accuracy with which the center axis of the transducer can be aligned with the center of the electrode recording area. We found that a short shift (≤1 mm) in the distance between the two centers led to different ultrasound pressure distributions (Figure 12A). Therefore, pressure measurements will be conducted before ultrasound stimulation in future procedures. Furthermore, we hope to significantly reduce the measurement time with respect to hydrophone sensitivity in a recording area over a 2D plain.

## Conclusion

Here, we constructed an MEA-based recording system combined with an ultrasound transducer and custom-designed waveguide to record activity in a brain slice elicited by an ultrasound short oscillation. We first conducted a numerical analysis to test an ultrasound stimulation model with a waveguide for mechanical stimulation of brain slices *in vitro*. We next designed and produced the waveguide on the basis of the numerical results, and then evaluated parameters for stimulation with the waveguide according to neural data recorded in the mouse auditory cortex on an MEA substrate. We were able to obtain the appropriate waveguide structure to produce the desired intensity distribution in a brain slice on an MEA substrate. Our experimental results also indicate that ultrasound-evoked cortical responses in mouse brain slices depend on the intensities and durations of the ultrasound stimulation. Furthermore, our analysis of the similarity between current- and ultrasound-driven response patterns indicated that the laminar properties of cortical slices and/or spatial patterns of ultrasound stimulations might influence ultrasound-driven response patterns. We successfully constructed an *in vitro* experimental setup for directly examining the possibility of locally activating a brain slice *via* ultrasound. Our MEA-based recording system has future potential for examining the detailed mechanisms of ultrasound stimulation of the brain.

## Data Availability Statement

The original contributions presented in the study are included in the article/Supplementary Material, further inquiries can be directed to the corresponding author.

## Ethics Statement

The animal study was reviewed and approved by the Institutional Animal Care and Use Committee of Hokkaido University.

## Author Contributions

RF and HK performed the experiments with the help of TT. RF and TT analyzed the data and wrote the manuscript in consultation with HK. All authors contributed to the design and implementation of the research and the numerical simulations.

## Conflict of Interest

The authors declare that the research was conducted in the absence of any commercial or financial relationships that could be construed as a potential conflict of interest.

## Publisher’s Note

All claims expressed in this article are solely those of the authors and do not necessarily represent those of their affiliated organizations, or those of the publisher, the editors and the reviewers. Any product that may be evaluated in this article, or claim that may be made by its manufacturer, is not guaranteed or endorsed by the publisher.

## References

[B1] Bachtold M. R. Rinaldi P. C. Jones J. P. Reines F. Price L. R. (1998). Focused ultrasound modifications of neural circuit activity in a mammalian brain. *Ultrasound Med. Biol.* 24 557–565. 10.1016/s0301-5629(98)00014-3 9651965

[B2] Bian T. Meng W. Qiu M. Zhong Z. Lin Z. Zou J. (2021). Noninvasive Ultrasound Stimulation of Ventral Tegmental Area Induces Reanimation from General Anaesthesia in Mice. *Research* 2021:2674692. 10.34133/2021/2674692 33954291 PMC8059556

[B3] Blackmore J. Shrivastava S. Sallet J. Butler C. R. Cleveland R. O. (2019). Ultrasound Neuromodulation: a Review of Results, Mechanisms and Safety. *Ultrasound Med. Biol.* 45 1509–1536. 10.1016/j.ultrasmedbio.2018.12.015 31109842 PMC6996285

[B4] Bystritsky A. Korb A. S. Douglas P. K. Cohen M. S. Melega W. P. Mulgaonkar A. P. (2011). A review of low-intensity focused ultrasound pulsation. *Brain Stimul.* 4 125–136. 10.1016/j.brs.2011.03.007 21777872

[B5] Cardenas-Rojas A. Pacheco-Barrios K. Giannoni-Luza S. Rivera-Torrejon O. Fregni F. (2020). Noninvasive brain stimulation combined with exercise in chronic pain: a systematic review and meta-analysis. *Exp. Rev. Neurother.* 20 401–412. 10.1080/14737175.2020.1738927 32130037 PMC7245447

[B6] Chew W. C. Liu Q. H. (1996). Perfectly matched layers for elastodynamics: a new absorbing boundary condition. *J. Comp. Acoust.* 4 341–359. 10.1142/s0218396x96000118

[B7] Choi J. B. Lim S. h. Cho K. w. Kim D. h. Jang D. p. Iy K. (2013). “The effect of focused ultrasonic stimulation on the activity of hippocampal neurons in multi-channel electrode,” in *International IEEE/EMBS Conference on Neural Engineering*, (New York, NY), 731–734.

[B8] Duck F. A. (2007). Medical and non-medical protection standards for ultrasound and infrasound. *Prog. Biophys. Mol. Biol.* 93 176–191. 10.1016/j.pbiomolbio.2006.07.008 16965806

[B9] Duda R. O. Hart P. E. Stork D. G. (2001). *Pattern classification.* New York, NY: Wiley.

[B10] Egert U. Heck D. Aertsen A. (2002). Two-dimensional monitoring of spiking networks in acute brain slices. *Exp. Brain Res.* 142 268–274. 10.1007/s00221-001-0932-5 11807580

[B11] Franklin K. Paxinos G. (2008). *The Mouse Brain in Stereotaxic Coordinates.* New York, NY: Academic Press.

[B12] Fregni F. Pascual-Leone A. (2007). Technology insight: noninvasive brain stimulation in neurology-perspectives on the therapeutic potential of rTMS and tDCS. *Nat. Clin. Pract. Neurol.* 3 383–393. 10.1038/ncpneuro0530 17611487

[B13] Guo H. Hamilton, Ii M. Offutt S. J. Gloeckner C. D. Li T. (2018). Ultrasound Produces Extensive Brain Activation via a Cochlear Pathway. *Neuron* 99:866. 10.1016/j.neuron.2018.07.049 30138592

[B14] Khraiche M. L. Phillips W. B. Jackson N. Muthuswamy J. (2008). Ultrasound induced increase in excitability of single neurons. *Annu. Int. Conf. IEEE Eng. Med. Biol. Soc.* 2008 4246–4249. 19163650 10.1109/IEMBS.2008.4650147

[B15] Kim H. B. Swanberg K. M. Han H. S. Kim J. C. Kim J. W. Lee S. (2017). Prolonged stimulation with low-intensity ultrasound induces delayed increases in spontaneous hippocampal culture spiking activity. *J. Neurosci. Res.* 95 885–896. 10.1002/jnr.23845 27465511

[B16] Kubanek J. Shukla P. Das A. Baccus S. A. Goodman M. B. (2018). Ultrasound Elicits Behavioral Responses through Mechanical Effects on Neurons and Ion Channels in a Simple Nervous System. *J. Neurosci.* 38 3081–3091. 10.1523/JNEUROSCI.1458-17.2018 29463641 PMC5864152

[B17] Liu Y. J. Wang G. Cao C. Zhang G. R. Tanzi E. B. Zhang Y. (2021). Neuromodulation Effect of Very Low Intensity Transcranial Ultrasound Stimulation on Multiple Nuclei in Rat Brain. *Front. Aging Neurosci.* 2021:13. 10.3389/fnagi.2021.656430 33935688 PMC8081960

[B18] Lochab J. Singh V. R. J. I. J. O. P. Physics A. (2004). Acoustic behaviour of plastics for medical applications. *Acoust. behav. plast. med. appl.* 42 595–599.

[B19] Menz M. D. Oralkan O. Khuri-Yakub P. T. Baccus S. A. (2013). Precise neural stimulation in the retina using focused ultrasound. *J. Neurosci.* 33 4550–4560. 10.1523/JNEUROSCI.3521-12.2013 23467371 PMC6704938

[B20] Menz M. D. Ye P. Firouzi K. Nikoozadeh A. Pauly K. B. Khuri-Yakub P. (2019). Radiation Force as a Physical Mechanism for Ultrasonic Neurostimulation of the Ex Vivo Retina. *J. Neurosci.* 39 6251–6264. 10.1523/JNEUROSCI.2394-18.2019 31196935 PMC6687898

[B21] Muramatsu S. Toda M. Nishikawa J. Tateno T. (2019). Sound- and current-driven laminar profiles and their application method mimicking acoustic responses in the mouse auditory cortex in vivo. *Brain Res.* 1721:146312. 10.1016/j.brainres.2019.146312 31323198

[B22] Namikawa M. Sano A. Tateno T. (2017). Salicylate-Induced Suppression of Electrically Driven Activity in Brain Slices from the Auditory Cortex of Aging Mice. *Front. Aging Neurosci.* 9:395. 10.3389/fnagi.2017.00395 29311894 PMC5732918

[B23] Pasquinelli C. Hanson L. G. Siebner H. R. Lee H. J. Thielscher A. (2019). Safety of transcranial focused ultrasound stimulation: a systematic review of the state of knowledge from both human and animal studies. *Brain Stimul.* 12 1367–1380. 10.1016/j.brs.2019.07.024 31401074

[B24] Prieto M. L. Firouzi K. Khuri-Yakub B. T. Maduke M. (2018). Activation of Piezo1 but Not NaV1.2 Channels by Ultrasound at 43 MHz. *Ultrasound Med. Biol.* 44 1217–1232. 10.1016/j.ultrasmedbio.2017.12.020 29525457 PMC5914535

[B25] Rinaldi P. C. Jones J. P. Reines F. Price L. R. (1991). Modification by focused ultrasound pulses of electrically evoked responses from an in vitro hippocampal preparation. *Brain Res.* 558 36–42. 10.1016/0006-8993(91)90711-4 1933382

[B26] Sato T. Shapiro M. G. Tsao D. Y. (2018). Ultrasonic Neuromodulation Causes Widespread Cortical Activation via an Indirect Auditory Mechanism. *Neuron* 98:1031. 10.1016/j.neuron.2018.05.009 29804920 PMC8127805

[B27] Szymanski F. D. Rabinowitz N. C. Magri C. Panzeri S. Schnupp J. W. (2011). The laminar and temporal structure of stimulus information in the phase of field potentials of auditory cortex. *J. Neurosci.* 31 15787– 15801. 22049422 10.1523/JNEUROSCI.1416-11.2011 PMC6623019

[B28] Toda M. Tateno T. (2020). Numerical Optimization of Waveguide Structure in an Ultrasound Brain Stimulation System Using theFDTDMethod. *Ieej Trans. Elect. Electr. Eng.* 15 1246–1247.

[B29] Tyler W. J. Tufail Y. Finsterwald M. Tauchmann M. L. Olson E. J. Majestic C. (2008). Remote excitation of neuronal circuits using low-intensity, low-frequency ultrasound. *PLoS One* 3:e3511. 10.1371/journal.pone.0003511 18958151 PMC2568804

[B30] Yamamura D. Sano A. Tateno T. (2017). An analysis of current source density profiles activated by local stimulation in the mouse auditory cortex in vitro. *Brain Res.* 1659 96–112. 10.1016/j.brainres.2017.01.021 28119054

[B31] Zecchi K. A. Mosgaard L. D. Heimburg T. R. (2017). Mechano-capacitive properties of polarized membranes and the application to conductance measurements of lipid membrane patches. *J. Phys.* 780:012001. 10.1088/1742-6596/780/1/012001 26324950

