# Supplementary material for: A Multielectrode Array-Based Recording System for Analyzing Ultrasound-Driven Neural Responses in Brain Slices in vitro
Source: Front Neurosci. 2022 Feb 22;16:824142. doi: 10.3389/fnins.2022.824142 (PMC8902160; doi:10.3389/fnins.2022.824142)
Supplement: Supplementary file 1 [file Data_Sheet_1.zip › Furukawa_FiNS_20220204/FileList_SupplementaryMaterial.pdf]

File list for supplementary material 2022.01.24

Item No.	File name	Format	Content
1	SupplementaryFigures_20220125.pdf	Portable document format (PDF)	Supplementary figures 1 to 4 and their legends
2	SupplementaryTables_20220125.pdf	Portable document format (PDF)	Supplementary tables 1 to 3
3	CADimage1_Waveguide.jpg	JPG image file	3D CAD image of a waveguide for a 3D printer
4	CADimage2_transducer_base.jpg	JPG image file	3D CAD image of a transducer base for a 3D printer
5	CADimage3_MEA_stage.jpg	JPG image file	3D CAD image of a MEA stage for a 3D printer
6	Presentation_COMSOL_setup_20220202.pdf	Portable document format (PDF)	Brief explanation of the COMSOL multipysics (mph) file
7	MED system_waveguide_config.mph	COMSOL maltiphysics MPH file format	Computational model setup and analysis script
8	File list	Portable document format (PDF)	This file
