# Supplementary material for: A Multielectrode Array-Based Recording System for Analyzing Ultrasound-Driven Neural Responses in Brain Slices in vitro
Source: Front Neurosci. 2022 Feb 22;16:824142. doi: 10.3389/fnins.2022.824142 (PMC8902160; doi:10.3389/fnins.2022.824142)

Ultrasound Simulation in an MEA-based Recording System

A Multielectrode Array-based Recording System for Analyzing Ultrasound-driven Neural Responses in Brain Slices In Vitro

Ryo Furukawa, Hiroki Kaneta, and Takashi Tateno

Outline

In COMSOL multiphysics software, an MPH file is the default standard file with the extension ".mph".

The files contain binary and text data. The mesh and solution data are usually stored as binary data, while all other information is stored as plain text.

In our setup file (“MED system_waveguide_config.mph”), the mesh and solution data are removed because of the large file size, so that they must be recalculated.

To compute the data, on the left tab, select “Model Builder >> Study 2” and, on the middle tab, “Settings >> Compute”, please.

In the following slides, our model parameters are briefly summarized. In the last slide, one example of our results is illustrated.

Geometry

- Models
  - Air
  - Water (ACSF) in the chamber
  - MEA (chamber and substrate)
  - Brain slice
  - Waveguide
  - Transducer (100 kPa)

Geometry

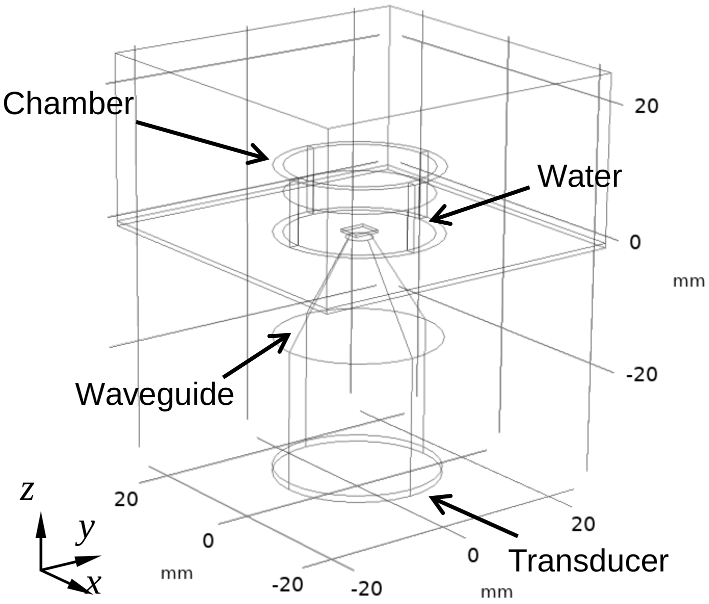

- Mesh size: extra fine
  - Maximum: 1.23 mm
  - Minimum: 0.123 mm
  - Mesh size “normal” would be better to obtain results on a personal computer

- Variable number: height of water in the chamber
  - Maximum: 9 mm
  - Minimum: 3 mm

Geometry: Size and Shape

- Air (block)
  - Width: 50 mm
  - Depth: 50 mm
  - Height: 25 mm

- Water in the chamber (cylinder)
  - Radius: 11 mm
  - Height: variable

- MEA chamber (cylinder)
  - Radius: 11 mm (inner), 12.5 mm (outer)
  - Height: 10 mm

Geometry: Size and Shape

- MEA substrate (block)
  - Width: 50 mm
  - Depth: 50 mm
  - Height: 0.7 mm

- Brain slice (block)
  - Width: 4 mm
  - Depth: 3.5 mm
  - Height: 0.4 mm

- Water in the waveguide 1 (cone)
  - Radius: 12.5 mm (bottom), 2 mm (top)
  - Height: 15 mm

- Water in the waveguide 2 (cylinder)
  - Radius: 12.5 mm
  - Height: 20 mm

Model Setup

- Sound soft boundary
  - The outer boundaries of the air domain
  - The boundary between the water and air

- Sound hard boundary
  - The outer boundaries of the MEA substrate domain
  - The outer boundaries of the waveguide domain

- Study: frequency domain

Acoustic impedance of each material

Material	Acoustic impedance (Pa·s/m)
Glass	15.58 M
Brain	1558 k
Air	409.7
Water	1480 k

Result

Example of the acoustic pressure results:

On the left tab, select Model Builder >> Results >> Export >> Plot 3.

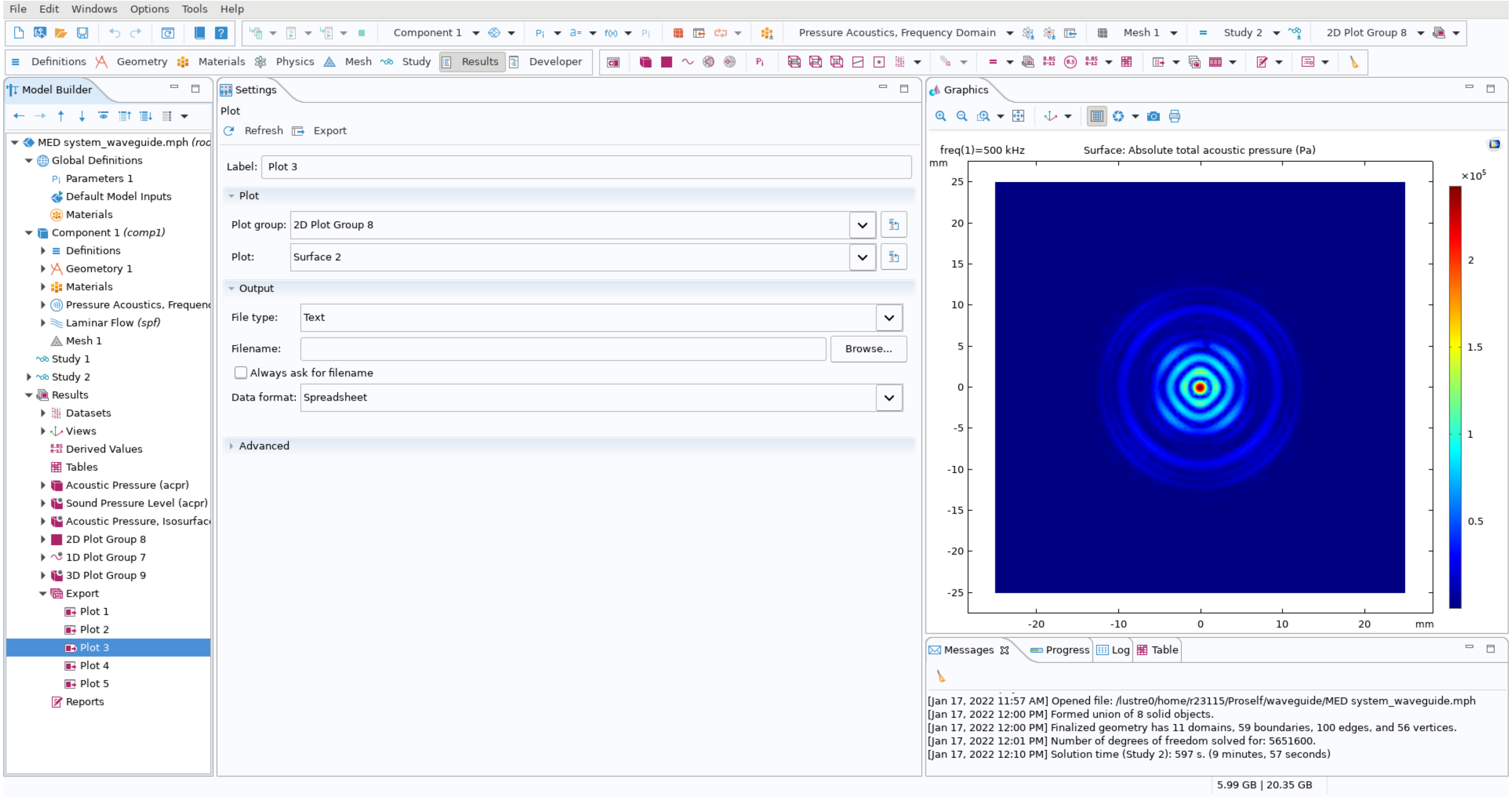

Supplement: Supplementary file 1 [file Data_Sheet_1.zip › Furukawa_FiNS_20220204/Presentation_COMSOL_setup_20220202.pdf]
