# Supplementary material for: A Multielectrode Array-Based Recording System for Analyzing Ultrasound-Driven Neural Responses in Brain Slices in vitro
Source: Front Neurosci. 2022 Feb 22;16:824142. doi: 10.3389/fnins.2022.824142 (PMC8902160; doi:10.3389/fnins.2022.824142)
Supplement: Supplementary file 1 [file Data_Sheet_1.zip › Furukawa_FiNS_20220204/SupplementaryFigures_20220125.pdf]

frontiers

Supplementary Figure legends

Figure 1. Local field potential (LFP) responses driven by ultrasound stimulation (intensity, 310 kPa; duration, 100 ms) before and after the application of 1-μM TTX in the ACSF solution. (A) Illustration showing examples of typical LFP response waveforms before and after the TTX application in (a) and (b), respectively. In (b), the waveform was obtained 30 min after the application. US-driven LFP responses were completely blocked. Bars over the waveforms indicate the duration of the ultrasound (US) stimulation. (B) Summary for negative-going peak intensities in all LFP responses driven by ultrasound stimulation. The result was obtained from five slices (n =5) with different animals. Thirty min after the TTX application, LFP responses were significantly reduced; the mark ** in the plot represents $P < 0.01$ for the paired t-test. In the plot, dots indicate individual means for each slice, and squares represent group means. Each error bar represents the standard error of the mean (SEM).

Figure 2. Similarity between cluster sets with respect to the response patterns driven by electrical stimulation. (A) A heatmap representing the normalized similarity of the cluster set, using a Euclidean measure of distance (dissimilarity). To calculate similarity values in the heatmap, we determined Euclidean distances (d_{euc}) between the centroids in the individual clusters. The similarity values (I_{sim}) are defined as $I_{sim} = (1 - d_{norm})$, where d_{norm} is the normalized value of d_{euc} with respect to the maximum for all distances. Numbers in the dendrogram represent the corresponding distance values between the two linked clusters. (B) A heatmap representing the normalized similarity of the set, using a cosine measure (normalized inner product of two vectors).

Figure 3. Similarity between a cluster set with respect to the response patterns driven by ultrasound stimulation. (A) A heatmap representing the normalized similarity of the cluster set, using a Euclidean measure of distance (dissimilarity). To calculate the similarity values in the heat map, we determined Euclidean distances (d_{euc}) between the centroids in the individual clusters. The similarity values (I_{sim}) are defined as $I_{sim} = (1 - d_{norm})$, where d_{norm} is the normalized value of d_{euc} with respect to the maximum for all distances. Numbers in the dendrogram represent the corresponding distance values between the two linked clusters. (B) A heatmap representing the normalized similarity of the set, using a cosine measure (normalized inner product of two vectors).

Figure 4. Numerically simulated patterns of ultrasound pressure distributions and normalized cross correlation with the cluster set induced by electrical stimulation. (A) Illustration showing 49 (7 × 7) patterns of numerically simulated pressure distributions. Each pattern was calculated from a pressure distribution in which the center position (x_0, y_0) of the ultrasound transducer was simply shifted by a step of 0.5 mm relative to the center of the MEA substrate. For the pattern labeled “25”, the center position was located at the origin of the MEA substrate: (x_0, y_0) = (0, 0). (B) Heatmap representing the correlation coefficients between the simulated pressure patterns and the representative patterns of the clusters associated with the responses driven by electrical stimulation. Dendrogram 1 from Fig. 11 is plotted again.

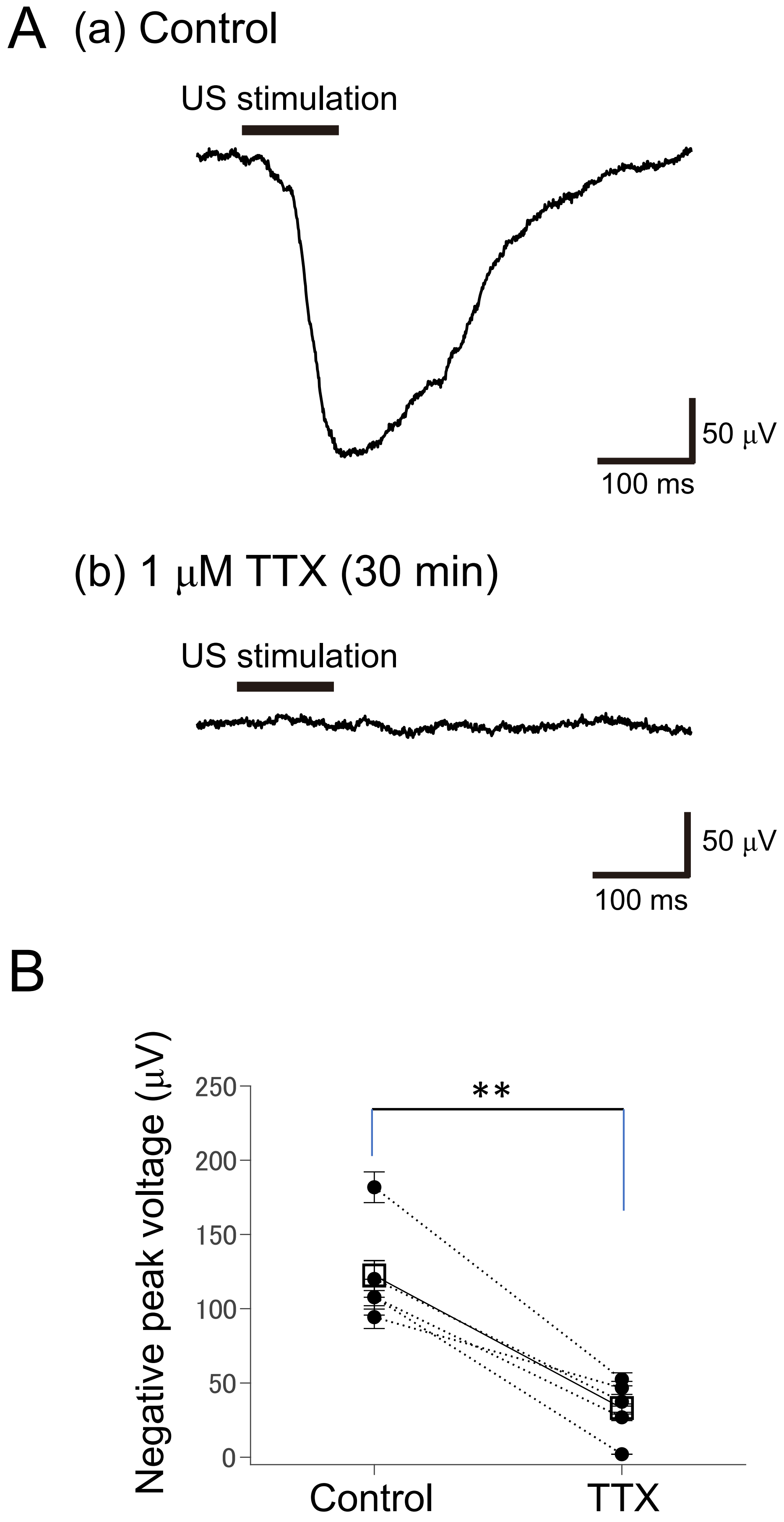

Supplementary Figure 1, Furukawa et al., 2022.01.15

Current-driven patterns: Clusters 1 to 7

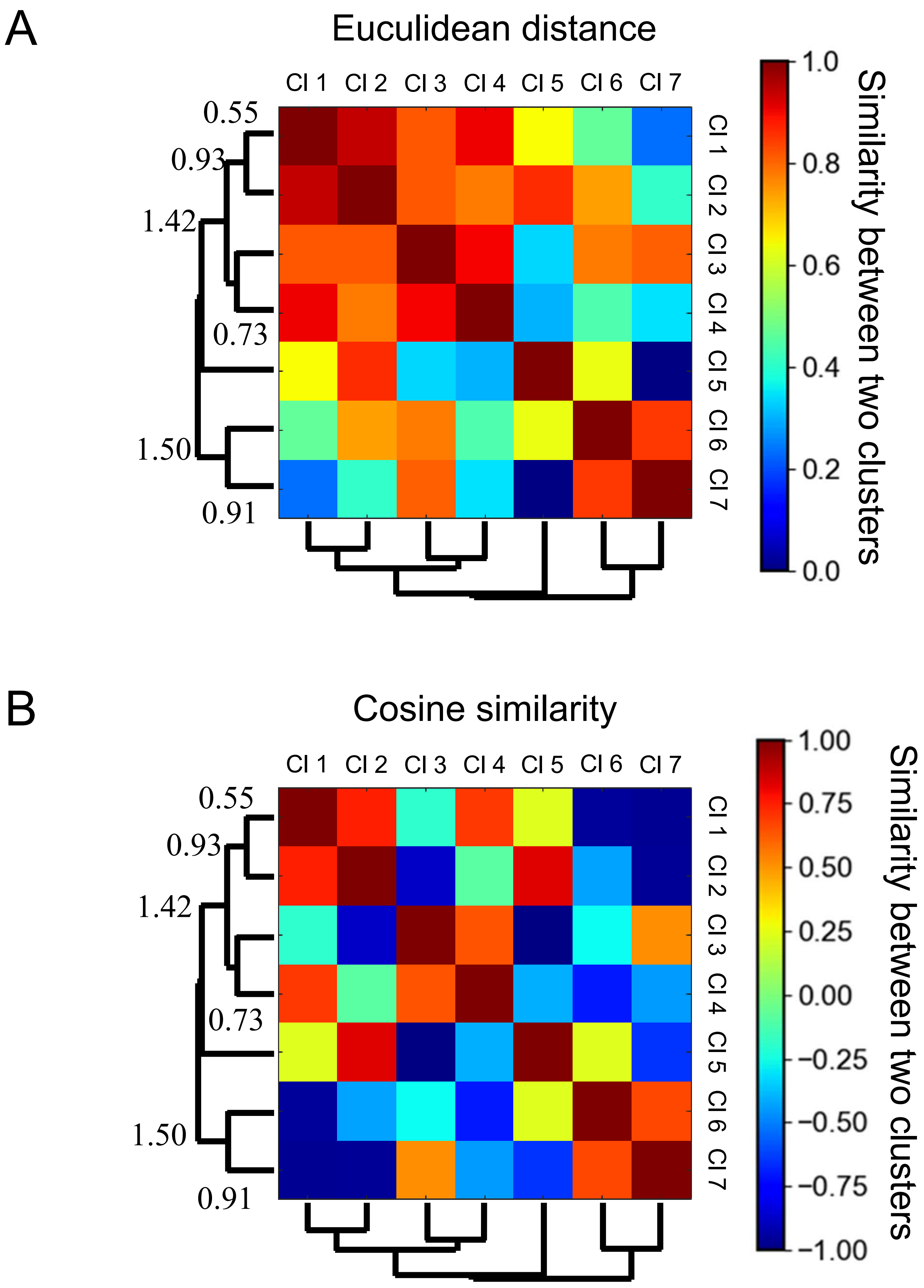

Supplementary Figure 2, Furukawa et al., 2022.01.17

Ultrasound-driven patterns: Clusters 1 to 5

A

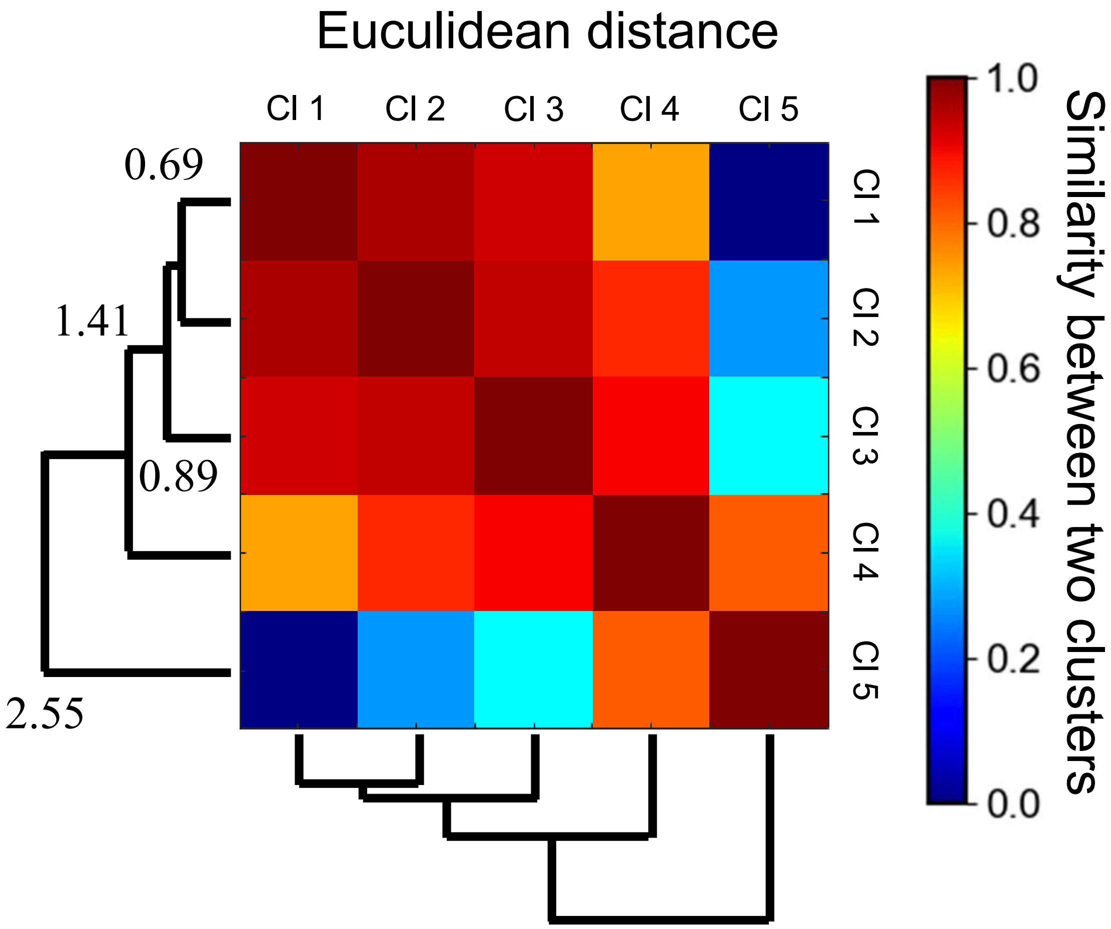

B

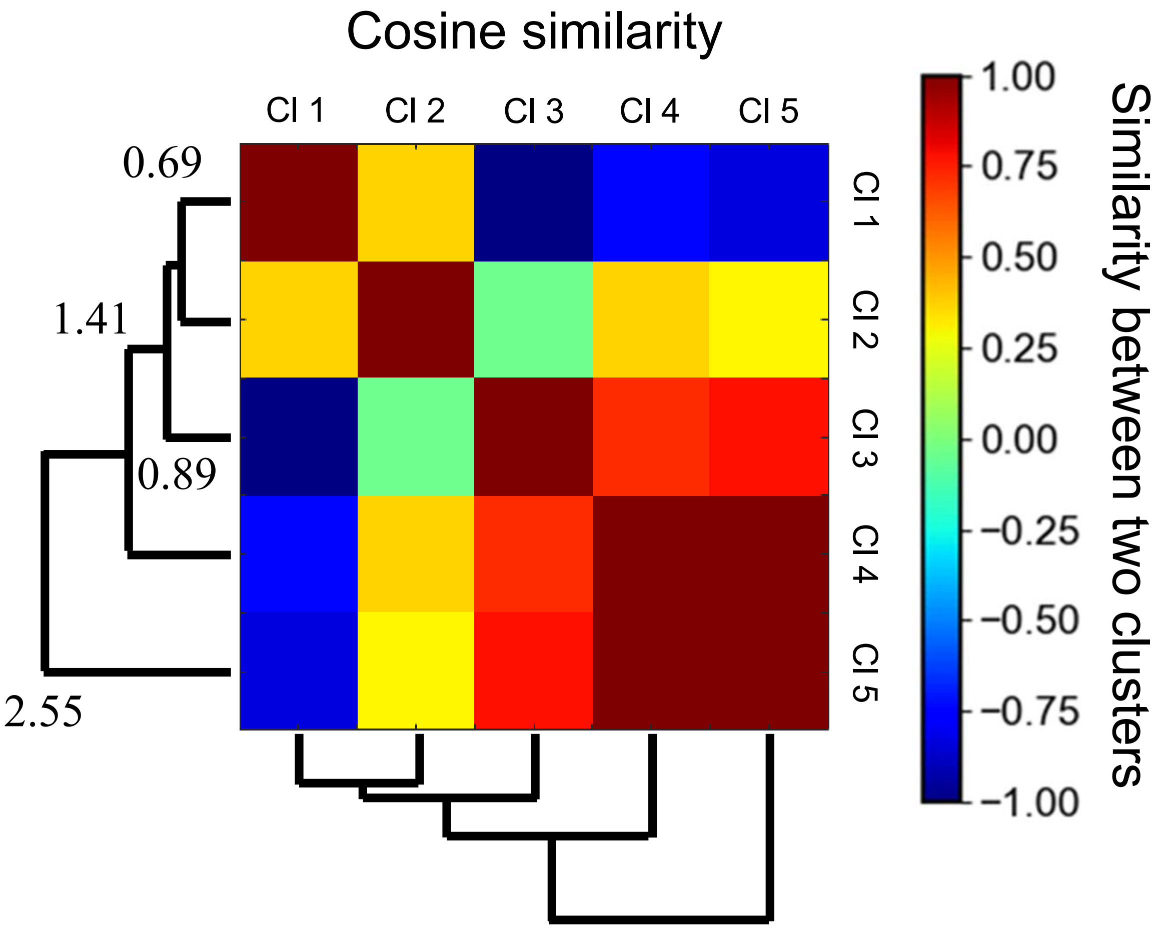

A

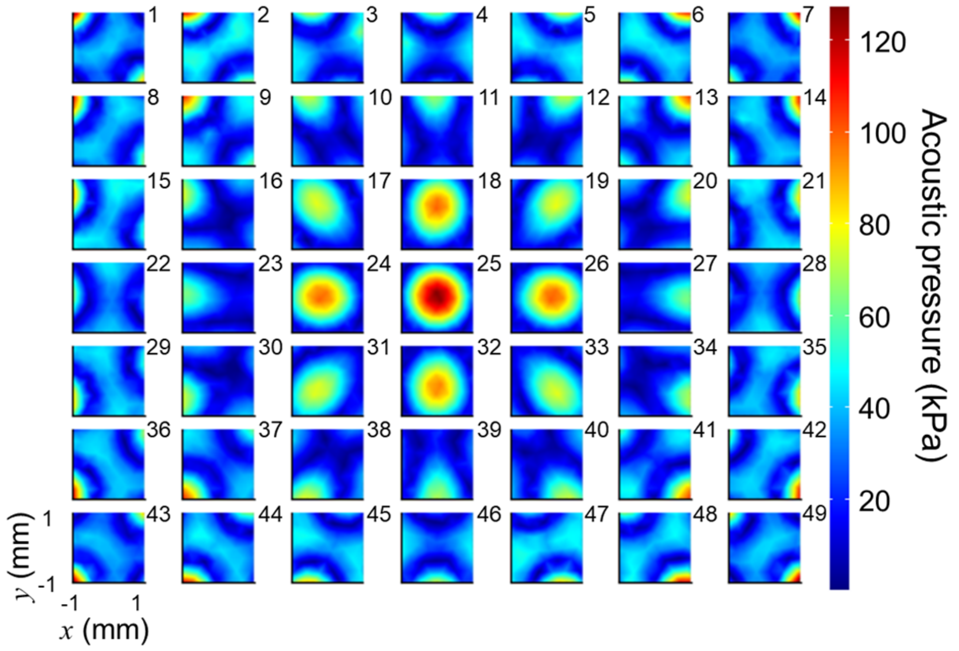

B

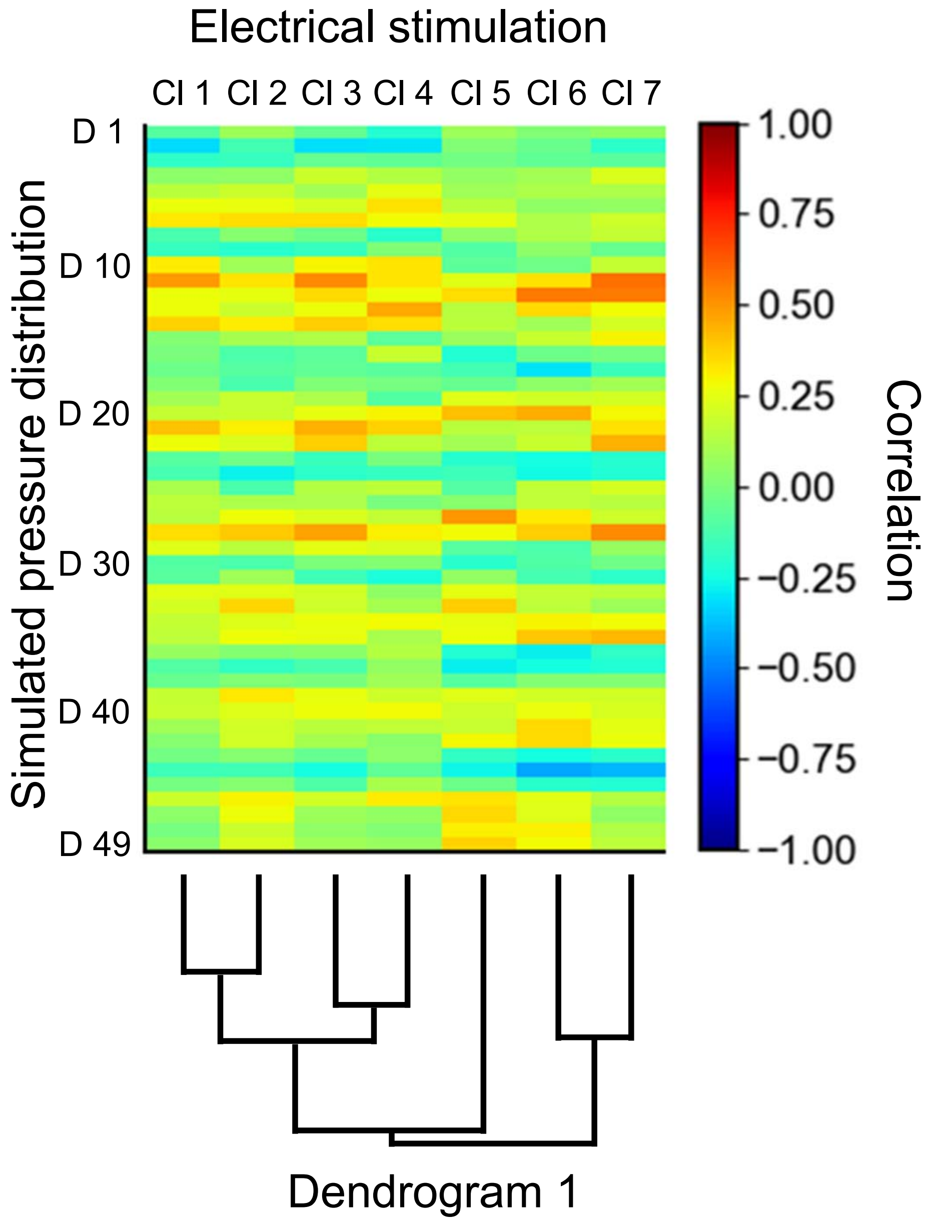

Supplementary Figure 4, Furukawa et al., 2022.01.17
