# Supplementary material for: A Multielectrode Array-Based Recording System for Analyzing Ultrasound-Driven Neural Responses in Brain Slices in vitro
Source: Front Neurosci. 2022 Feb 22;16:824142. doi: 10.3389/fnins.2022.824142 (PMC8902160; doi:10.3389/fnins.2022.824142)
Supplement: Supplementary file 1 [file Data_Sheet_1.zip › Furukawa_FiNS_20220204/SupplementaryTables_20220125.pdf]

Supplementary Table 1. Summary of sample size for clusters 1 to 7 in response to current stimulation

Slice No.	Animal No.	Sample size							Total size
		Cluster 1	Cluster 2	Cluster 3	Cluster 4	Cluster 5	Cluster 6	Cluster 7	
1	1 (#20210106)	0	0	0	0	159	0	0	159
2	2 (#20201223)	3	0	0	157	0	0	0	160
3	2 (#20201223)	0	0	0	0	0	0	160	160
4	3 (#20200918)	0	0	0	0	0	260	0	260
5	4 (#20200624)	0	0	160	20	0	0	0	180
6	5 (#20200131)	0	60	0	0	0	0	0	80
7	5 (#20200131)	90	0	0	0	0	0	0	90
Total size		93	60	160	177	159	260	160	1069

Supplementary Table 2. Summary of sample size for clusters 1 to 5 in response to ultrasound stimulation

Slice No.	Animal No.	Sample size					Total size
		Cluster 1	Cluster 2	Cluster 3	Cluster 4	Cluster 5	
1	1 (#20210106)	3	0	0	3	1	7
2	2 (#20201223)	5	0	0	0	0	5
3	2 (#20201223)	1	0	0	0	0	1
4	3 (#20200918)	2	0	58	2	0	62
5	4 (#20200624)	8	0	0	32	30	70
6	5 (#20200131)	44	126	0	3	3	176
7	5 (#20200131)	30	0	0	0	0	30
Total size		93	126	58	40	34	351

Supplementary Table 3. Comparison of previous studies associated with ultrasound stimulation to in-vitro brain preparations

Authors, year	Preparation	Carrier frequency in MHz	US stimulation pattern	Intensity in mW/cm^2 (pressure level in kPa)
Tyler et al., 2008	Hippocampal slices and isolated mouse brain	0.44	Repetitive tone burst	I_{SPTA}, 2,900
Khraiche et al., 2008	Dissociated cultures from Hippocampus	7.55	Repetitive tone burst	I_{SPTA}, 50,000 – 150,000
Menz et al., 2013 & 2019	Isolated salamander retina	0.50 – 43	Repetitive tone burst	I_{SPTA}, 10,000 – 40,000
Choi et al., 2013	Rat hippocampal neurons	0.50	Repetitive tone burst	$I_{SPTA,}$ 16.1 – 92.8
Kim et al., 2017	Hippocampal slices	0.50	Repetitive tone burst	I_{SPTA}, 0.78 (11.5)
Prieto et al., 2018	Cell cultures	43	Sinusoidal wave pulse	I_{SPPA}, 50,000 – 90,000
Kubanek et al., 2018	Caenorhabditis Elegans	10	Repetitive tone burst	I_{SPTA} < 1,000
Current study, 2021	Mouse acute cortical slices	0.50	Sinusoidal wave pulse	I_{SPPA}, 310 – 3,750 (< 410)
