# Supplementary figures and images for: A Multielectrode Array-Based Recording System for Analyzing Ultrasound-Driven Neural Responses in Brain Slices in vitro
Source: Front Neurosci. 2022 Feb 22;16:824142. doi: 10.3389/fnins.2022.824142 (PMC8902160; doi:10.3389/fnins.2022.824142)

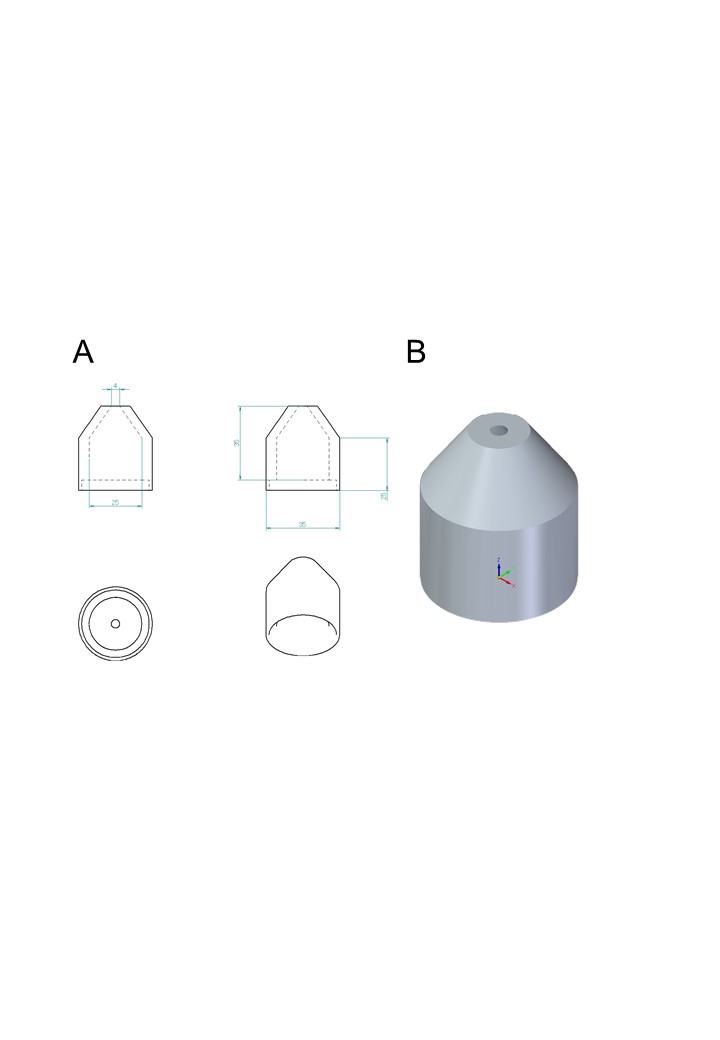

Supplement: Supplementary file 1 [file Data_Sheet_1.zip › Furukawa_FiNS_20220204/CADimage1_Waveguide.jpg]

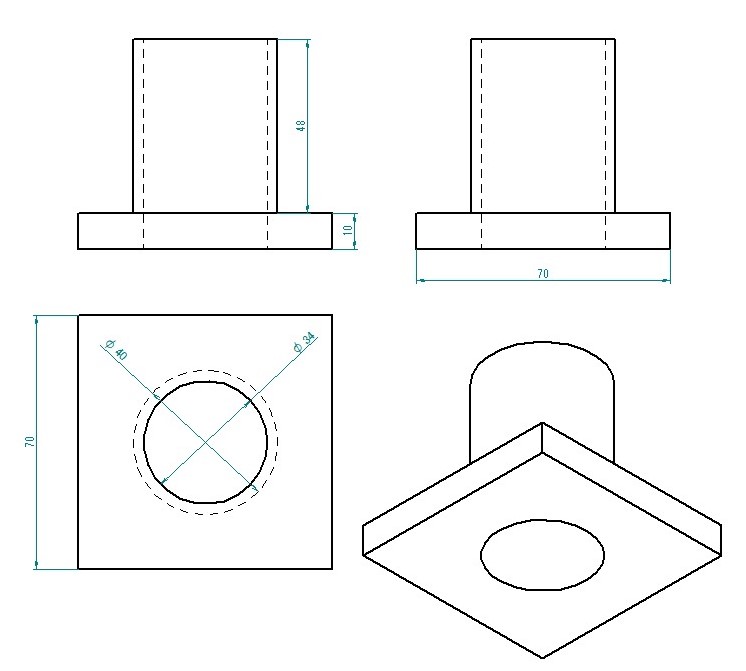

Supplement: Supplementary file 1 [file Data_Sheet_1.zip › Furukawa_FiNS_20220204/CADimage2_transducer_base.jpg]

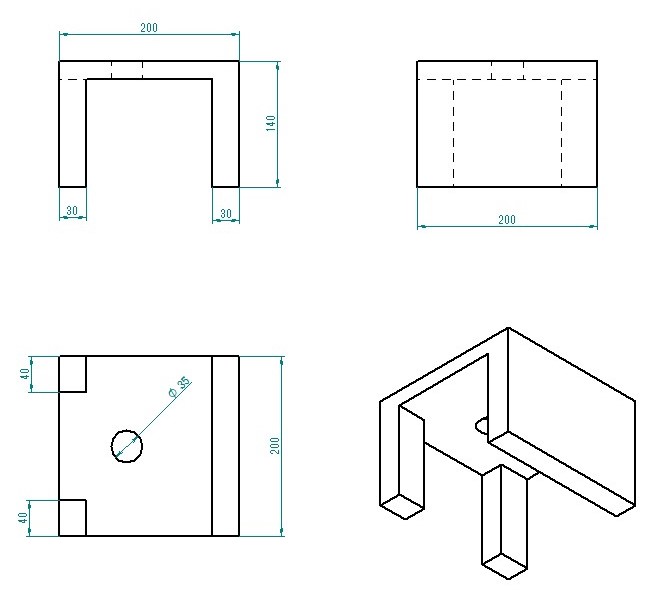

Supplement: Supplementary file 1 [file Data_Sheet_1.zip › Furukawa_FiNS_20220204/CADimage3_MEA_stage.jpg]
